# The Different Roles of *Penicillium oxalicum* LaeA in the Production of Extracellular Cellulase and β-xylosidase

**DOI:** 10.3389/fmicb.2016.02091

**Published:** 2016-12-22

**Authors:** Yanan Li, Xiaoju Zheng, Xiujun Zhang, Longfei Bao, Yingying Zhu, Yinbo Qu, Jian Zhao, Yuqi Qin

**Affiliations:** ^1^State Key Lab of Microbial Technology and National Glycoengineering Research Center, Shandong UniversityJinan, China; ^2^Shandong Provincial Key Laboratory of Carbohydrate Chemistry and Glycobiology, Shandong UniversityJinan, China

**Keywords:** cellulase, β-xylosidase, LaeA, transcription factor, *Penicillium oxalicum*

## Abstract

Cellulolytic enzyme hydrolysis of lignocellulose biomass to release fermentable sugars is one of the key steps in biofuel refining. Gene expression of fungal cellulolytic enzymes is tightly controlled at the transcriptional level. Key transcription factors such as activator ClrB/CLR2 and XlnR/XYR1, as well as repressor CreA/CRE1 play crucial roles in this process. The putative protein methyltransferase LaeA/LAE1 has also been reported to regulate the gene expression of the cellulolytic enzyme. The formation and gene expression of the cellulolytic enzyme was compared among *Penicillium oxalicum* wild type (WT) and seven mutants, including Δ*laeA* (deletion of *laeA*), OE*clrB* (*clrB* overexpression), OE*clrB*Δ*laeA* (*clrB* overexpression with deletion of *laeA*), OE*xlnR* (*xlnR* overexpression), OE*xlnR*Δ*laeA* (*xlnR* overexpression with deletion of *laeA*), Δ*creA* (deletion of *creA*), and Δ*creA*Δ*laeA* (double deletion of *creA* and *laeA*). Results revealed that LaeA extensively affected the expression of glycoside hydrolase genes. The expression of genes that encoded the top 10 glycoside hydrolases assayed in secretome was remarkably downregulated especially in later phases of prolonged batch cultures by the deletion of *laeA*. Cellulase synthesis of four mutants Δ*laeA*, OE*clrB*Δ*laeA*, OE*xlnR*Δ*laeA*, and Δ*creA*Δ*laeA* was repressed remarkably compared with their parent strains WT, OE*clrB*, OE*xlnR*, and Δ*creA*, respectively. The overexpression of *clrB* or *xlnR* could not rescue the impairment of cellulolytic enzyme gene expression and cellulase synthesis when LaeA was absent, suggesting that LaeA was necessary for the expression of cellulolytic enzyme gene activated by ClrB or XlnR. In contrast to LaeA positive roles in regulating prominent cellulase and hemicellulase, the extracellular β-xylosidase formation was negatively regulated by LaeA. The extracellular β-xylosidase activities improved over 5-fold in the OE*xlnR*Δ*laeA* mutant compared with that of WT, and the expression of prominent β-xylosidase gene *xyl3A* was activated remarkably. The cumulative effect of LaeA and transcription factor XlnR has potential applications in the production of more β-xylosidase.

## Introduction

Biological conversion of cellulosic biomass to fuels and chemicals through enzymatic hydrolysis of cellulose allows the production of substantial amounts of biofuels and biochemicals to replace fossil fuels. The enzymatic hydrolysis of lignocellulose biomass to release fermentable sugars is one of the key steps in biofuel refining (Himmel and Bayer, 2009). Many filamentous fungi are able to degrade plant cell wall material by secreting large numbers of cell wall-degrading enzymes, including cellulase and hemicellulase (Lynd et al., 2002). Therefore, investigating the regulation of lignocellulosic enzyme gene expression is important to improve the production of these enzymes in biofuel refining.

Mechanisms of lignocellulosic enzyme gene regulation have been extensively studied in filamentous fungi such as *Trichoderma reesei* (teleomorph *Hypocrea jecorina*) (Seiboth et al., 2004; Kubicek et al., 2009), *Aspergilli* (Gielkens et al., 1999), and *Neurospora crassa* (Coradetti et al., 2012). Fungal cellulase and hemicellulase gene expression are tightly controlled at the transcriptional level (Ilmén et al., 1997). The expression of most cellulase and hemicellulase genes is transcriptionally co-regulated by the coordinated action of transcription factors. Several transcription factors have been identified to play essential roles in the expression of cellulase in *T*. *reesei, Aspergillus nidulans, N*. *crassa*, and *Penicillium oxalicum*, such as the repressor CRE1/CreA (Strauss et al., 1995) and ACE1 (Aro et al., 2003) and the positive regulators XYR1/XlnR and CLR2/ClrB (Coradetti et al., 2012; Li et al., 2015).

CRE1 (ortholog is CreA in *Aspergillus* spp.) is the key transcription factor found in filamentous fungi. Increased cellulase production was observed in the CRE1 deletion/mutation strains of *T. reesei* (Nakari-Setälä et al., 2009) and *N*. *crassa* (Sun and Glass, 2011). In *T. reesei*, CRE1 binding sites were found in the prominent cellulase gene *cbh1* and prominent hemicellulase gene *xyn1* promoters, where mutations in the binding sequences led to the constitutive expression of these genes even in the presence of glucose (Ilmén et al., 1996; Mach et al., 1996). XYR1 (ortholog is XlnR in *Aspergillus* spp.) is a general activator of hydrolase formation in many fungi. In *T. reesei* and *Aspergilli*, XYR1/XlnR has been shown to positively affect the transcription of major cellulase and hemicellulase genes (van Peij et al., 1998; Stricker et al., 2006). The overexpression of the *xlnR* gene in *Aspergillus oryzae* has resulted in elevated cellulolytic and xylanolytic activities in the culture supernatant (Noguchi et al., 2009). Recently, a zinc binuclear cluster transcription factor CLR-2 was reported to be an important activator of both cellulase and hemicellulase genes in *N. crassa* (Coradetti et al., 2012). CLR-2 is conserved in the genomes of most filamentous fungi. In *A*. *nidulans*, approximately 50% of the cellulose-responsive genes showed strict dependence on functional *clr-2* homolog (*clrB*), and the cellulolytic activity on Avicel was significantly impaired as a result of *clrB* deletion (Coradetti et al., 2013).

In addition to the key transcription factors, other regulators such as LAE1 were also found to play important roles in regulating cellulase and hemicellulase gene expression. The *lae1*/*laeA* (*l*oss of *a*flR *e*xpression) gene was originally identified in *A. nidulans* (Bok et al., 2005). This gene was later proven to affect morphological development and the biosynthesis of a large number of secondary metabolites in many filamentous fungi, such as *Aspergilli* (Kale et al., 2008; Oda et al., 2011; Chang et al., 2012), *Penicillium chrysogenum* (Kosalková et al., 2009), and *Fusarium verticillioides* (Butchko et al., 2012). LaeA's function is always associated to the epigenetic control by its putative protein methyltransferase function (specifically for histone tails), as LaeA possesses S-adenosyl-methionine-binding (SAM) motifs. However, LaeA's direct methyl-accepting substrate has not been discovered yet, although it was shown to be self-methylated in Met 207 (Patananan et al., 2013). LaeA somehow is assumed to counteract the trimethylation of H3K9 and the binding of heterochromatin protein to this repressive chromatin mark (Strauss and Reyes-Dominguez, 2011). LaeA was also found to control glycoside hydrolase gene and transcriptional activator Xyr1 gene expression in *T. reesei*. A complete loss of expression of all seven cellulases and auxiliary factors for cellulose degradation and low *xyr1* transcript levels was observed in *T. reesei laeA* deletion strain (Seiboth et al., 2012).

*Penicillium oxalicum*, previously called *Penicillium decumbens*, has been used for industrial-scale glycoside hydrolase production for more than 20 years in China (Qu et al., 1991). Data from the entire genome-sequencing analysis revealed that this fungus has a unique lignocellulose-degrading enzyme arsenal during evolution, including 18 predicted cellulase genes, 51 predicted hemicellulase genes, and 12 additional genes that encode accessory enzymes for the degradation of cellulose and hemicellulose (Liu et al., 2013a). The mechanism behind the regulation of lignocellulosic enzyme genes in *P. oxalicum* is similar to that in *Aspergillus* and *Neurospora*. Several transcription factors such as CreA (Liu et al., 2013a), ClrB, and XlnR (Li et al., 2015) have been found to play important roles in regulating cellulolytic enzyme gene expression. For instance, the overexpression of *clrB*, combined with both deletion of *creA* and an intracellular β-glucanse gene *bgl2*, remarkably activated the cellulase gene expression and improved filter paper activity (FPA) by over 20-fold (Li et al., 2015; Yao et al., 2015).

In this study, we investigated the roles of *P. oxalicum* LaeA in regulating the expression of cellulase and β-xylosidase gene, especially when transcription activator gene (*clrB* or *xlnR*) was overexpressed or transcription repressor gene (*creA*) was deleted. We found that LaeA extensively affected *P*. *oxalicum* glycoside hydrolase gene expression, especially in later phases of prolonged batch cultures. The transcription repressor gene *creA* was also affected by LaeA. The overexpression of transcription activator gene *clrB* or *xlnR* could not rescue the impairment of cellulolytic enzyme gene expression and cellulase synthesis when LaeA was absent. Interestingly, in contrast to the remarkable downregulation of prominent cellulase and hemicellulase by the deletion of *laeA*, the extracellular β-xylosidase formation was an exception. The extracellular β-xylosidase activities improved over 5-fold in the OE*xlnR*Δ*laeA* mutant compared with that of WT, and the expression of prominent β-xylosidase gene *xyl3A* was activated remarkably.

## Materials and methods

### Strains and culture conditions

All the strains used in this study were listed in Table 1. Fungal strains were routinely grown on potato dextrose agar (PDA) or Vogel's medium agar (Vogel, 1956), which were supplemented or modified as indicated. For conidial production, PDA plates were incubated at 30°C for 5 days and aerial conidia were harvested by flooding the plate with sterile distilled H_2_O containing 0.02% Tween 80. Conidial concentrations were determined by direct count using a hemocytometer.

**Table 1:** **Strains of *P. oxalicum* used in this study**.

**Strains**	**The description of genotype**	**Source or references**
WT	Wild type 114-2	This lab
Δ*laeA*	Δ*laeA*::*ptra*	Zhang et al., 2016
R*laeA*	Δ*laeA*::*ptra hph::laeA*	Zhang et al., 2016
OE*laeA*	*hph*::P*gpdA*::*laeA*	This study
OE*clrB*	*hph*::P*gpdA*::*clrB*	Li et al., 2015
OE*clrB*Δ*laeA*	*hph*::P*gpdA*::*clrB* Δ*laeA*::*ptra*	This study
OE*xlnR*	*hph*::P*gpdA*::*xlnR*	Li et al., 2015
OE*xlnR*Δ*laeA*	*hph*::P*gpdA*::*xlnR* Δ*laeA*::*ptra*	This study
Δ*creA*	Δ*creA*::*hph*	Liu et al., 2013a
Δ*laeA*Δ*creA*	Δ*creA*::*hph* Δ*laeA*::*ptra*	Zhang et al., 2016

### Construction of mutants in *P. oxalicum*

Standard techniques were used for nucleic acid manipulations. An overlap PCR method (Davidson et al., 2002) was used to create the fragments for targeted gene deletion. *P. oxalicum* 114-2 genomic DNA was used as the template for the PCR amplification of the 5′- and 3′-flanking regions of the *laeA*. The plasmid pME2892 (Krappmann et al., 2005) was used as the template for the PCR amplification of resistant gene pyrithiamine (*ptrA*). Primers UlaeA-F/UlaeA-R, ptrA-F/ptrA-R, and DlaeA-F/DlaeA-R were usded to amplify the 5′ homologous flanking region (2296 bp), *ptrA* gene (2008 bp), and 3′ homologous flanking region (3072 bp) of *laeA*, respectively. The three resulting PCR fragments were fused and amplified by PCR using the nested primers ClaeA-F/ClaeA-R. The PCR products were directly transformed into OE*clrB* or OE*xlnR* to obtain OE*clrB*Δ*laeA* or OE*xlnR*Δ*laeA*. The primers hyg-F and hyg-R were used to amplify the *hph* gene (a hygromycin B phosphotransferase encoding gene (*hphG*, 2425 bp) from plasmid pSilent1 (Nakayashiki et al., 2005). The primers gpdA-F and gpdA-LaeA-R were used to amplify the glyceraldehydes-3-phosphate dehydrogenase promoter (*gpdA*) from *A*. *nidulans* genome (1256 bp), whereas the primers LaeA-F/LaeA-R were used to amplify the intact *P. oxalicum laeA* open reading frame with its terminator (2179 bp). The three resulting PCR fragments were fused and transformed into WT to obtain OEΔ*laeA*.

The protoplast preparation, transformation, and screening of recombinant clones were performed as previously described by Qin et al. (2013). The genomic DNA of the transformants was isolated and used as the template to verify the integration events. Primers YlaeA-1/ptrA-R and YlaeA-2/ptrA-F were used to certify the deletion of *laeA* and obtained 2840 bp and 2413 bp fragments, respectively. Primers Yhyg-gpdA-F/Yhyg-gpdA-R and YgpdA-F/YgpdA-laeA-R were used to certify the overexpression of *laeA* and obtained 2263 bp and 1299 bp fragments, respectively. Then, the mutants were further analyzed by Southern blot. Primers S-DlaeA-F/S-DlaeA-R and were used for PCR amplification to generate the probes for the examination of OE*clrB*Δ*laeA* and OE*xlnR*Δ*laeA*. Primers S-OElaeA-F and S-OElaeA-R were used for PCR amplification to generate the probes for the examination of OE*laeA*. The probes were labeled via PCR using a PCR DIG Probe Synthesis kit (Roche). The genomics DNA were digested with the restriction endonucleases and separated from 0.75% agarose gel electrophoresis. Then the DNA was transferred to the Hybond-N + nylon membranes (Amersham Biosciences/GE Healthcare, USA). Blots were visualized using a DIG DNA labeling system following the manufacturer's protocol (Roche). The PCR verification and Southern blot results are shown in Figure S1. The primers used in this study were listed in Table S1.

### Phenotypic analysis of wild type and mutants

One microliter conidial suspension (1 × 10^6^ conidia ml^−1^) of each strain (1 μL hyphal fragment solution of Δ*creA*Δ*laeA* strain was dropped onto the agar because it cannot generate conidia) were spotted onto various media on a 9-cm plate, including PDA or modified Vogel's salts agar medium (Vogel, 1956) with various carbon sources supplemented with 2% (W/V) glucose, 2% lactose, 2% glycerol, or 2% ball-milled microcrystalline cellulose at 30°C for 4 days. Canon EOS 600D (Canon, Japan) was used for photographing. To test for conidiation, 10^6^ conidia were spread on 9-cm Vogel's + glucose plates and were cultivated at 30°C for 4 days. Afterward, a 5-mm diameter colony agar plug was removed from the plate. Three replications were conducted for each treatment. Conidia were then harvested by gently rubbing them off in an equal volume of physiological salt (0.2% w/v Tween 80 and 0.8% w/v NaCl). Conidial concentrations were determined by direct count using a hemocytometer.

### Cellulase and xylosidase activity assay

The strains were first grown in 100 mL liquid glucose minimal medium (GMM, per liter: glucose 20.0 g, KH_2_PO_4_ 3 g, (NH4)_2_SO_4_ 2.0 g, MgSO_4_·7H_2_O 0.56 g, CaCl_2_ 0.56 g, FeSO_4_·7H_2_O 7.5 mg, MnSO_4_·H_2_O 2.5 mg, ZnSO_4_·7H_2_O 3.6 mg, CoCl_2_·6H_2_O 3.7 mg, CuSO_4_ 3.2 mg) with initial pH 5.5 at 200 rpm for 24 h in 30°C. Then, 0.3 g vegetative mycelia was collected through vacuum filtration and was added to 100 mL of Vogel's salts medium supplemented with 20 g L^−1^ inducing carbon sources (10.0 g L^−1^ microcrystalline cellulose plus 10.0 g L^−1^ wheat bran) at 30°C and 200 rpm. Samples were collected at the time points indicated in the text. Microcrystalline cellulose (CB0279) was purchased from Sangon (Shanghai, China). Culture supernatants were diluted with sodium acetate buffer solution (0.2 M, pH 4.8). The filter paper activities (FPA) of the culture supernatants were assayed against Whatman No. 1 filter paper using a DNS method (Sun et al., 2008). β-D-xylopyranoside (p-NPX) was used as the substrate for β-xylosidase activity assay. The experiment was conducted by incubating 50 μL of 0.1 mg/mL p-NPX in a 0.1 M sodium citrate buffer (pH 4.8) with 100 μL enzyme elute for 30 min at 50°C. Then, 150 μL of 10% (w/v) Na_2_CO_3_ was added to terminate the reaction. One enzyme activity unit was defined as the amount of enzymes required to produce 1 μmol glucose or pNP per minute under the assayed conditions. Three biological triplicates were performed in all analyses.

### SDS-PAGE and biomass analysis

Unconcentrated supernatants were added to loading buffer, boiled for 5 min for degeneration, and loaded onto a 12% Tris-HCl polyacrylamide gel. Coomassie blue stain reagent was used for staining. The biomasses of all mutants were measured using the methods described by Yao et al. (2015). Briefly, the WT and mutants were pre-grown in 1 × Vogel's medium with glucose for at 30, 200 rpm for 24 h. The same quantity of dehydrated mycelia from each strain was transferred to the same freshly prepared media for another 6, 12, 24, 36, 48, 60, 72, and 96 h. All sampled mycelia were dried at 65°C to achieve constant weight, and dry cell weight was estimated.

### Gene expression analysis by qRT-PCR

The strains were cultivated following the method described in “Cellulase and xylosidase activity assay.” Total RNA was extracted from frozen lyophilized mycelia using the RNAiso Plus reagent (TaKaRa Biotechnology). Then, RNA was treated with DNase I, and first- and second-strand cDNA synthesis was performed using a PrimeScript RT Reagent kit with gDNA Eraser (TaKaRa Biotechnology). qRT-PCR was performed by SYBR Premix Ex Taq (Perfect Real Time, TaKaRa Biotechnology) using a LightCycler 480 system with software version 4.0 (Roche, Mannheim, Germany). The primers that were used to examine gene expression levels included act-F/act-R (for the *actin* gene, EPS26156.1), cel7A-F/cel7A-R (for the *cel7A*/*cbh1* gene), cel7B-F/cel7B-R (for the *cel7B*/*eg1* gene), cel3A-F/cel3A-R (for *cel1A*/*bgl1* gene), amy15A-F/amy15A-R (for the *amy15A* gene), xln10A-F/xln10A-R (for *xln10A* gene), xyl3A-F/xyl3A-R (for *xyl3A* gene), laeA-F/laeA-R (for the *laeA* gene, EPS25650), creA-F/creA-R (for the *creA* gene, EPS28222), clrB-F/clrB-R (for the *clrB* gene, EPS31045), and xlnR-F/xlnR-R (for the *xlnR* gene, EPS32714). The primers used are shown in Table S1. Three biological triplicates were performed, and qRT-PCR of each gene was performed in three triplicates. The expression of actin was chosen as the reference gene for data normalization. The number of gene expression copies was calculated using the standard curves constructed for each gene, and the data were normalized to the expression levels of the actin gene. The relative expression level was defined as follows: Rel. expression level (gene X) = copy number of gene X/copy number of gene action. Statistical analysis was performed using Minitab, and *P* ≤ 0.05 were considered statistically significant. The primers used in this study were listed in Table S1.

### Digital gene expression profiling and GO analysis

The culture method was the same as that described in the “Cellulase and xylosidase activity assay” section. Digital gene expression profiling was performed according the methods described by Zhang et al. (2016). Briefly, after 24 h or 60 h of cultivation, total RNA was extracted from frozen mycelia using the RNAiso Plus reagent (TaKaRa Biotechnology). Oligo (dT) beads were employed to purify mRNA and guide double-stranded cDNA synthesis. Digital gene expression profiling based on Illumina sequencing was performed by the Beijing Genomics Institute in Shenzhen, China. Each tunnel generated millions of raw reads with a sequencing length of 35 bp. The 30 adaptor sequences were first removed from the sequencing reads to produce 21-nt long tags. Afterward, low quality tags, one copy tags, and tags which were not 21-nt long were removed to generate clean tags. All clean tags were obtained after raw data processing was mapped to the reference tag database (predicted transcripts plus downstream 300-nucleotide sequences) of the corresponding strain. The copy number of unambiguous tags (tags mapped to a single gene) for each gene was normalized to TPM for differential expression analysis. Genes with significantly different expression levels were identified through a significance test with combined thresholds (FDR ≤ 0.001 and fold change ≥ 2) (Audic and Claverie, 1997). Blast2GO was used for the function enrichment analysis of gene sets with the threshold at FDR ≤ 0.05 (Conesa et al., 2005).

### Yeast two-hybrid assay

Yeast two-hybrid assay was performed according the protocols of Clontech (Clontech Laboratories, Inc.). Briefly, pGBKT7 derived prey *laeA* and pGADT7 derived bait *creA, clrB* or *xlnR* were co-transformed into the *Saccharomyces cerevisiae* strain AH109, which has four reporter genes including *lacZ, mel1, ade2* and *his3*. Transformants were selected on SD/-Trp/-Leu media. To further confirm the interaction between LaeA and transcription factos, the transformants were tested on the SD/-Trp/-Leu/ containing 10 mg/ml X-α-gal. And the transforms were spotted in the SD/-Trp/-Leu/-His media containing 2.5 mM 3AT.

### Accession numbers

The Whole Genome Shotgun projects were deposited in DDBJ/EMBL/GenBank under the accession number AGIH00000000. The raw data of expression profiling were deposited in NCBI's Gene Expression Omnibus (GEO) database under the accession number GSE71287.

## Results

### All the mutants with the deletion of *laeA* showed less conidiation

**Figure 1:**
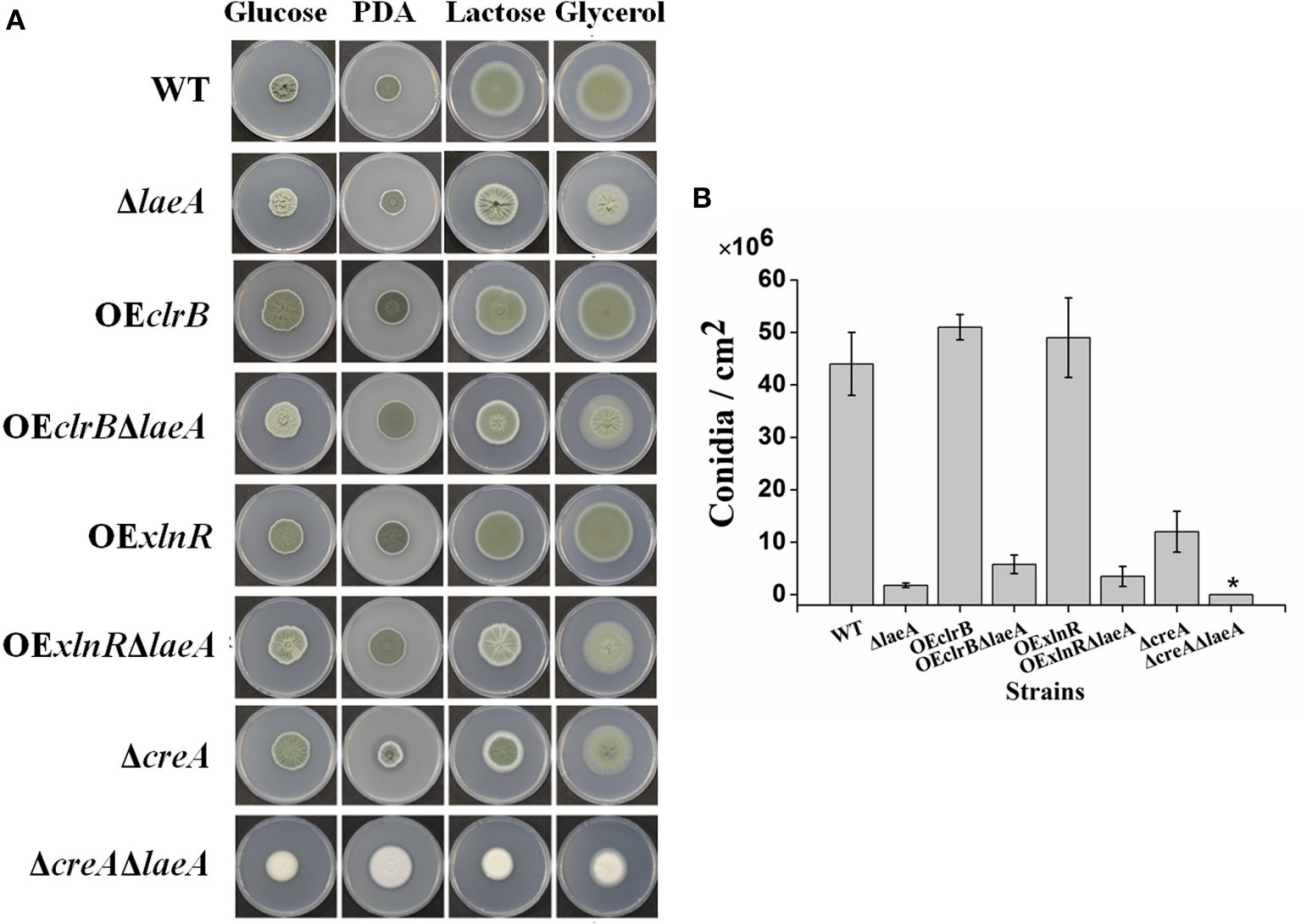
**Colony morphology and conidiation of WT and various mutants. (A)** Colony morphology of 4-day-old cultures on PDA or Vogel's medium agar with 2% glucose, 2% lactose, and 2% glycerol at 30°C. For WT, Δ*laeA*, OE*clrB*, OE*clrB*Δ*laeA*, OE*xlnR*, OE*xlnR*Δ*laeA*, and Δ*creA*, 1 μL conidia solution was dropped onto the agar at a density of 10^6^ conidia mL^−1^. For Δ*laeA*Δ*creA*, 1 μL of hyphal fragment solution was dropped onto the agar because it could not generate conidia. **(B)** Levels of conidiation on Vogel's medium agar with 2% glucose. Plates were incubated at 30°C for 4 days, and 5-mm diameter colony agar plugs in triplicate were sampled for each strain. The number of conidia was determined by using a hemocytometer. ^*^, no conidia were observed.

The fungal strains used in this study are listed in Table 1. Equivalent fresh spores of the WT and mutants were inoculated on PDA plates or on 1 × Vogel's salts (Vogel, 1956) with 2% (w/v) glucose, 2% lactose, 2% glycerol, or 2% cellulose as the sole carbon source for 4 days at 30°C (Figure 1A). Then, the colony morphology and the conidiation were observed. The colonies of Δ*laeA*, OE*clrB*Δ*laeA*, and OE*xlnR*Δ*laeA* appeared greenish-brown compared with the dark-green colonies observed in WT, OE*clrB*, and OE*xlnR*. Δ*creA*Δ*laeA* even formed a white colony (Figure 1A). Conidiation was quantified by plating conidia on top of agar layers, and conidiospore production was subsequently analyzed. The conidiation levels in all *laeA* deletion mutants were dramatically reduced; the conidiation of Δ*laeA*, OE*clrB*Δ*laeA*, and OE*xlnR*Δ*laeA* was ~4.1, ~13.2, and ~9.0% of WT, respectively (Figure 1B). No conidium was observed in Δ*creA*Δ*laeA*, consistent with previous report (Zhang et al., 2016). This observation indicated that LaeA positively regulated asexual development.

### All the mutants with the deletion of *laeA* showed reduced cellulase formation

**Figure 2:**
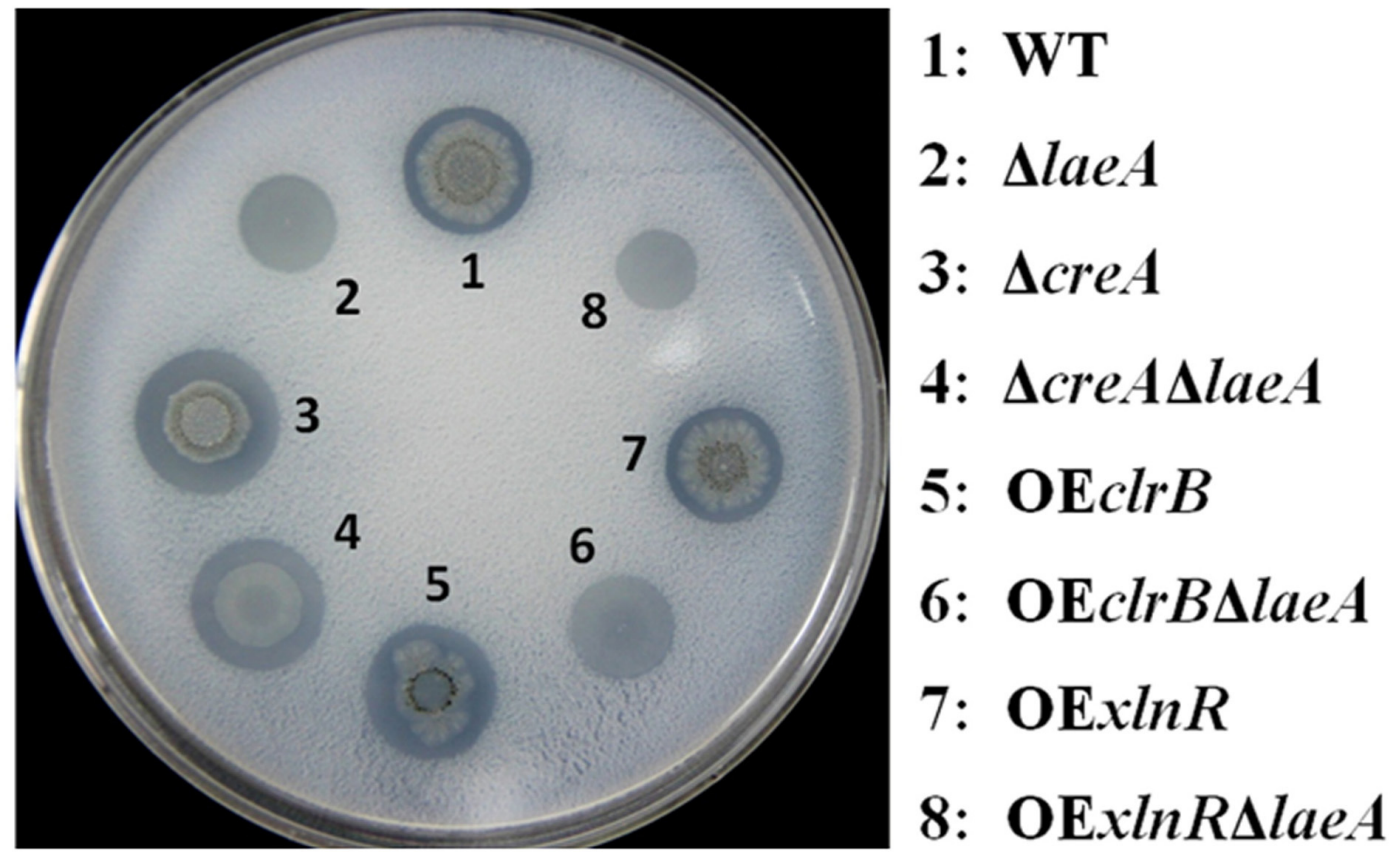
**Cellulolytic phenotype of WT and various mutants**. Colony morphology of 4-day-old cultures on Vogel's medium agar added with 2% cellulose at 30°C. For WT, Δ*laeA*, OE*clrB*, OE*clrB*Δ*laeA*, OE*xlnR*, OE*xlnR*Δ*laeA*, and Δ*creA*, 1 μL of conidia solution was dropped onto the agar at a density of 10^6^ conidia mL^−1^. For Δ*laeA*Δ*creA*, 1 μL of hyphal fragment solution was dropped onto the agar because it cannot generate conidia.

When the strains were grown on cellulose agar (1 × Vogel's salts plus with 2% cellulose as the sole carbon), the cellulolytic halo of all the four mutants Δ*laeA*, OE*clrB*Δ*laeA*, OE*xlnR*Δ*laeA*, and Δ*creA*Δ*laeA* decreased remarkably compared with their parent strains WT, OE*clrB*, OE*xlnR*, and Δ*creA*, respectively. No cellulolytic halo was observed around the Δ*laeA* colony, whereas a clear cellulolytic halo was found around the WT colony (Figure 2). ClrB and XlnR were transcription activators for the cellulase gene (Stricker et al., 2006; Coradetti et al., 2012); thus, it was expected their overexpression strain (OE*clrB* and OE*xlnR*) showed increased cellulolytic ability with a more pronounced cellulolytic halo than that of the WT; the result was consistent with the previous report described by Li et al. (2015). When *laeA* was deleted, however, the overexpression of *clrB* (OE*clrB*Δ*laeA*) or *xlnR* (OE*xlnR*Δ*laeA*) did not result in the formation of a cellulolytic halo (Figure 2). CreA suppressed the expression of most cellulase and hemicellulase genes through carbon catabolite repression mechanism (Nakari-Setälä et al., 2009). As a result, the deletion of *creA* (Δ*creA*) was observed, exhibiting a large and clear cellulolytic halo around its colony compared with that of the WT. The result was consistent with the previous report described by Liu et al. (2013a). However, when *laeA* was absent, the diameter of the cellulolytic halo around Δ*creA*Δ*laeA* was reduced (Figure 2).

**Figure 3:**
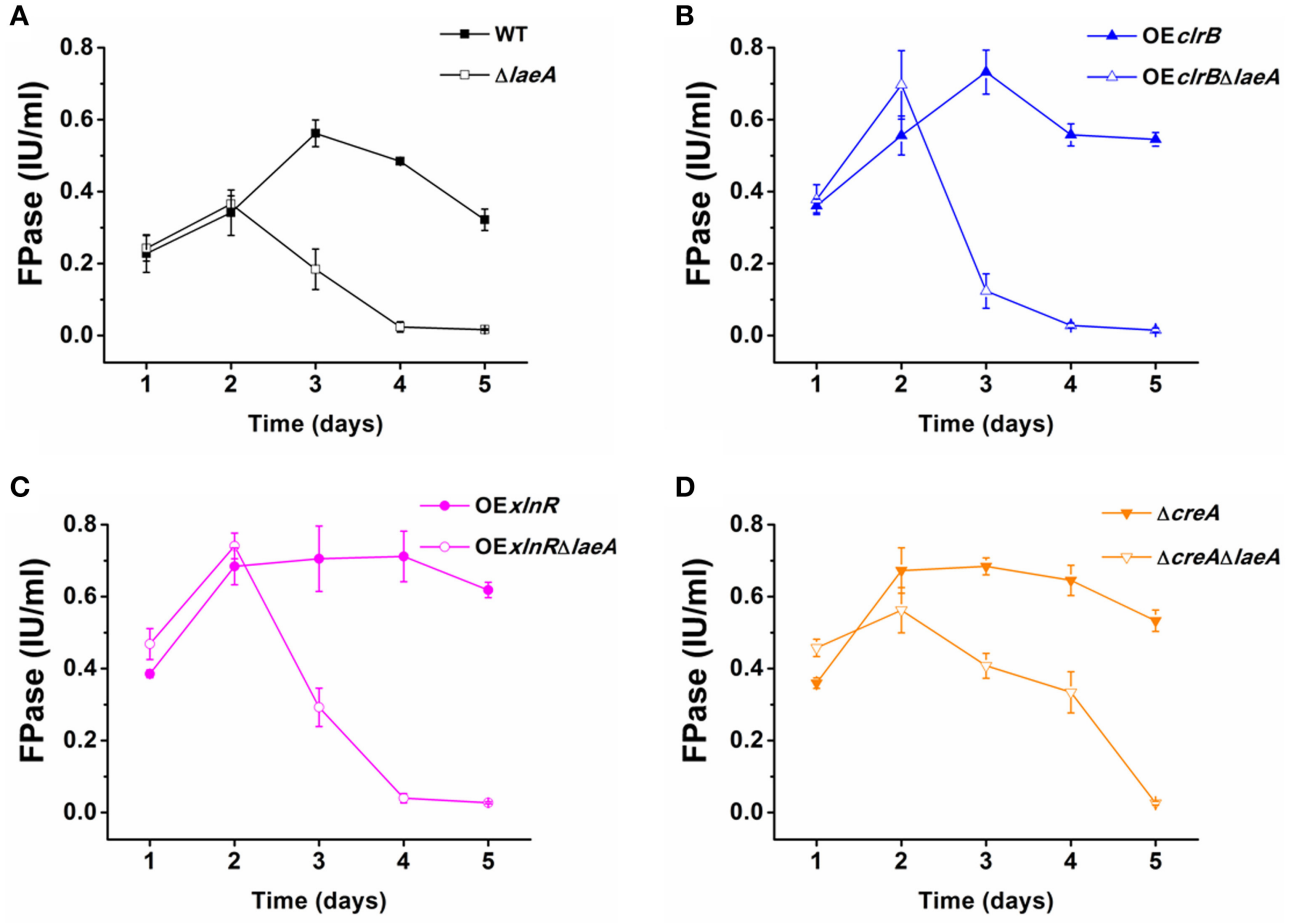
**Cellulolytic activity assay of WT and various mutants**. The strains were cultivated in liquid Vogel's salts medium supplemented with 1% wheat bran and 1% microcrystalline cellulose as carbon resources to induce cellulase gene expression. The strains were cultivated at 30°C for 5 days. FPA, a measure of the combined activities of both endo- and exo-type cellulases, was determined by hydrolyzing a strip of filter paper (Whatman #1, 50 mg, 1 cm × 6 cm) for 1 h at 50°C. **(A)** FPA of WT and Δ*laeA*. **(B)** FPA of OE*clrB* and OE*clrB*Δ*laeA*. **(C)** FPA of OE*xlnR* and OE*xlnR*Δ*laeA*. **(D)** FPA of Δ*creA* and Δ*creA*Δ*laeA*. The values show the mean of three biological replicates, and the error bar indicates the standard deviation.

For the agar, pure cellulose was used as the sole carbon source. Complex carbon sources from plant materials were more efficient than pure cellulose in promoting the expression of lignocellulose-degrading enzymes. Therefore, we cultivated the WT and the mutants in a submerged medium with wheat bran and microcrystalline cellulose. Then, the levels of FPA (representing overall cellulase activity) were assayed. To facilitate the comparison of FPA produced by different mutants, same scale of vertical ordinate was used in the Figure 3. It was expected that OE*clrB*, OE*xlnR*, and Δ*creA* showed increased FPA activities compared with WT. However, in the absence of LaeA, the FPA of all the four mutants Δ*laeA*, OE*clrB*Δ*laeA*, OE*xlnR*Δ*laeA*, and Δ*creA*Δ*laeA* decreased remarkably compared with their parent strains WT, OE*clrB*, OE*xlnR*, and Δ*creA*, respectively, after 2 days cultivation. On the third day, the FPA of Δ*laeA*, OE*clrB*Δ*laeA*, OE*xlnR*Δ*laeA*, and Δ*creA*Δ*laeA* was only 32.1, 16.4, 41.4, and 58.9%, respectively, compared with that of the their parent strain WT (Figure 3A), OE*clrB* (Figure 3B), OE*xlnR* (Figure 3C), and Δ*creA* (Figure 3D) strains. The FPA of OE*clrB*Δ*laeA*, OE*xlnR*Δ*laeA*, and Δ*creA*Δ*laeA* also decreased compared with that of 21.4, 56.8, and 73.2% WT, respectively. The difference between the mutants and WT increased along with prolonged culture time; the FPA of Δ*laeA*, OE*clrB*Δ*laeA*, OE*xlnR*Δ*laeA*, and Δ*creA*Δ*laeA* was only 5.0%, 8.1%, 4.7%, and 8.4%, respectively, compared with that of WT on the fifth day (Figure 3).

Then, the supernatants from the WT and the mutants were profiled by sodium dodecyl sulfate polyacrylamide gel electrophoresis (SDS–PAGE) (Figure S2). Equal volumes of supernatants were loaded. Significantly lesser protein bands were detected in Δ*laeA*, OE*clrB*Δ*laeA*, OE*xlnR*Δ*laeA*, and Δ*creA*Δ*laeA* than in WT, especially in the range of 40–116 kDa. Previous reports have shown that this range is an area for aggregation of glycoside hydrolases; in particular, most glucoamylases, cellobiohydrolases, endoglucanases, and β-glucosidases were located in the area (Liu et al., 2013b). Were the changes in cellulase synthesis in the mutants caused by their different biomass levels? The growth kinetics of WT and all the mutants were examined (Figure S3). Except that Δ*creA* and Δ*creA*Δ*laeA* exhibited lower biomass formation than WT, the similar growth kinetic curve of biomass level was observed among WT, Δ*laeA*, OE*clrB*, OE*xlnR*, OE*clrB*Δ*laeA*, and OE*xlnR*Δ*laeA*, showing that the reduced cellulolytic activities in Δ*laeA*, OE*clrB*Δ*laeA*, and OE*xlnR*Δ*laeA* or increased cellulolytic enzyme activities in OE*clrB* and OE*xlnR* did not correlate with the biomass level. The introduction of a wild type copy of *laeA* at the *laeA* locus (R*laeA*) restored the growth defects and the cellulolytic enzyme synthesis defects of the Δ*laeA* mutant (Figure S4). No evident difference was observed in the conidiation and cellulolytic enzyme synthesis in the mutant of overexpressed *laeA* (OE*laeA*) in *P. oxalicum* (Figure S5).

### LaeA extensively affected glycoside hydrolase gene expression, especially in later phases of prolonged batch cultures

We noticed that the deletion of *laeA* resulted in a massive reduction of FPA, especially in prolonged batch cultures (Figure 3). To obtain a global view of the effect of *laeA* deletion on glycoside hydrolase expression, the expression profiles of WT and Δ*laeA* were assayed. Glycoside hydrolases were characteristically produced after the shift from exponential growth to stationary phase. Therefore, two time points for gene expression profiling were selected. In particular, “24 h time point” indicates the exponential growth phase (earlier phases of cultures), whereas “60 h time point” indicates a stationary phase and exhibits a cellulase formation peak (later phases of prolonged batch cultures). WT and Δ*laeA* were cultivated in cellulose and wheat bran medium, which is an optimum medium for cellulolytic enzyme formation. The mRNA from WT and Δ*laeA* at the cultivation time of 24 h and 60 h, respectively, was collected and was subjected to high-throughput Illumina sequencing. More than 3 million tags were obtained for each sample. The copy number of unambiguous tags (tags mapped to one single gene) for each gene was normalized to the number of transcripts per million (TPM) clean tags. A genome-wide expression profiling analysis revealed the extensive expression of the entire *P. oxalicum* genome. Of the 10021 protein-coding genes in the genome database, 8192 (81.7%), 8292 (82.7%), 8351 (83.3%), and 8229 (82.1%) genes were expressed in WT-24 h, Δ*laeA*-24 h, WT-60 h, and Δ*laeA*-60 h, respectively. Genes of significantly differential expression levels were identified through a significance test with combined thresholds (FDR ≤ 0.001 and fold change ≥2). Blast2GO was used for function enrichment analysis of gene sets with a threshold of FDR ≤ 0.05.

The Δ*laeA* mutant was compared with WT at 24 h, showing that the expression levels of 1657 genes exhibited significant differences (2-fold or greater, FDR < 0.001) between Δ*laeA* and WT. Of these, 719 were regulated 4-fold or greater (FDR < 0.001), with 399 genes downregulated and 320 genes upregulated. GO enrichment analysis revealed that downregulated genes (4-fold or greater, FDR < 0.05) were mainly involved in heme binding, monooxygenase activity, catalytic activity, hydrolase activity, acting on glycosyl bonds, and binding of ACP phosphopantetheine attachment site binding involved in fatty acid biosynthetic process (Figure S6A). The upregulated genes were mainly involved in substrate-specific transmembrane transporter activity (Figure S6B). The downregulated and upregulated genes (GO category: molecular function), along with their predicted functions, are listed in Tables S2, S3.

For the Δ*laeA* mutant compared with WT at 60 h, the expression levels of 2488 genes exhibited significant differences (2-fold or greater, FDR < 0.001) between Δ*laeA* and WT. Of these, 1145 were regulated 4-fold or greater (FDR < 0.001), with 703 downregulated genes and 442 upregulated genes. GO enrichment analysis revealed that the downregulated genes (4-fold or greater, FDR < 0.05) in Δ*laeA* compared with that in WT were mainly involved in cellulose binding, aspartic-type endopeptidase activity, cellulase activity, monooxygenase activity, heme binding, electron carrier activity, and cellulose 1,4-beta-cellobiosidase activity (Figure S6A). GO enrichment analysis revealed no statistically significant results for the upregulated genes with this cutoff. The downregulated genes (GO category: molecular function) and their predicted functions are listed in Table S4.

**Figure 4:**
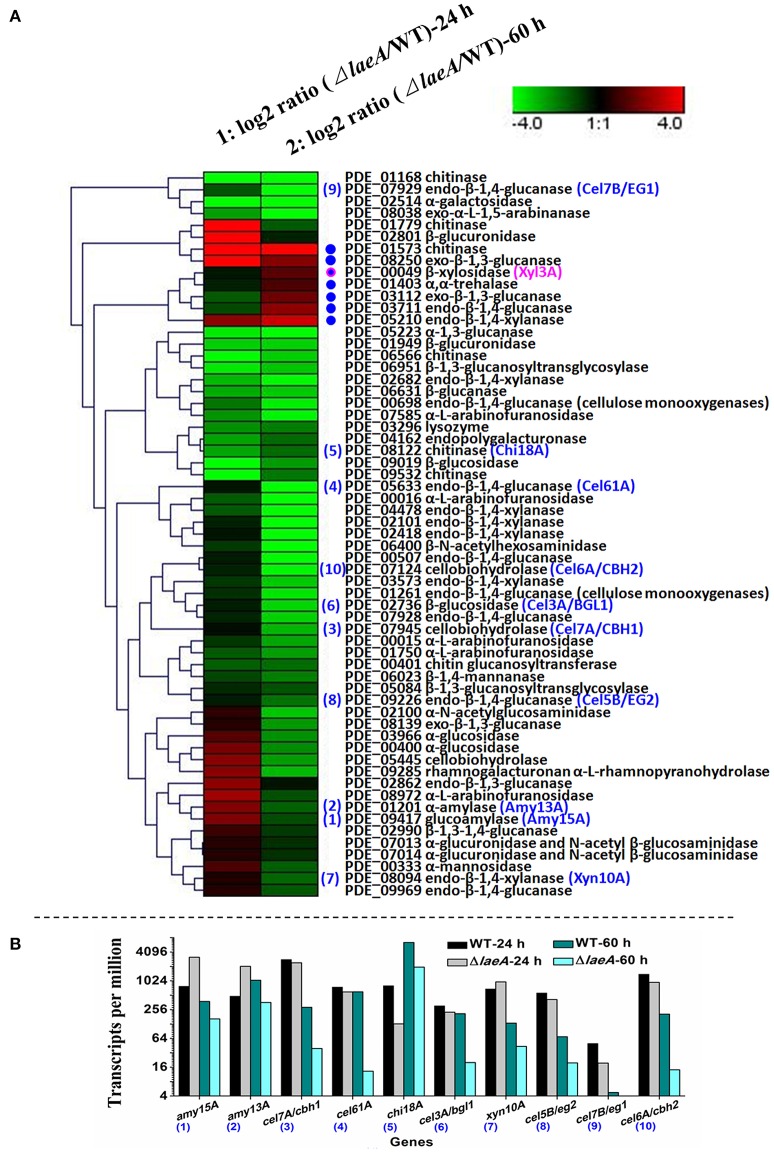
**Expression profiling analysis of glycoside hydrolase genes in Δ*laeA* compared with WT**. WT and Δ*laeA* were cultivated in cellulose and wheat bran medium, which are optimum media for glycoside hydrolase formation. mRNA from WT and Δ*laeA* at cultivation time of 24 and 60 h, respectively, was collected and subjected to high-throughput Illumina sequencing. **(A)** Expression analysis of 61 glycoside hydrolase genes in Δ*laeA* compared with WT at 24 and 60 h. The color of each block represents the log2 (fold change) in gene expression. The blue numbers from (1) to (10) (from the highest secretion amount to the lowest) represent the top 10 extracellular proteins found in *P. oxalicum* secretome (Liu et al., 2013b). The blue dots indicate seven genes, the expression levels of which are upregulated at 60 h. The blue dot with a pink circle indicates a β-xylosidase-encoding gene *xyl3A*. **(B)** Expression levels of the top 10 extracellular protein-encoding genes. The numbers (1) to (10) correspond to the numbers in **(A)**. The copy number of unambiguous tags (tags mapped to a single gene) for each gene was normalized to TPM (number of transcripts per million clean tags).

Of the downregulated genes, many secondary metabolism hallmark genes and glycoside hydrolase genes were present. As expected, many genes involved in secondary metabolism were downregulated by *laeA*, which agreed with previous reports (Palmer and Keller, 2010). We focused on the glycoside hydrolase gene expression. Among the 129 predicted glycosidase hydrolase genes expressed in the four samples, the expression levels of 61 genes (*P* < 0.001) were significantly different between Δ*laeA* and WT at both 24 h and 60 h (Figure 4A). Moreover, the gene expression of 40 (65.6%) glycosidase hydrolase was downregulated in Δ*laeA* compared with that in WT at the 24 h time point. Furthermore, the *laeA* exhibited a strong and wide-ranging effect on glycoside hydrolase transcriptional regulation observed at 60 h. The gene expression of 54 (88.5%) glycosidase hydrolase was significantly downregulated in Δ*laeA* compared with that in WT (Figure 4A).

When *P. oxalicum* was cultivated on cellulose and wheat-bran-containing media, the secretome data revealed that the top 10 extracellular proteins were glycoside hydrolases, including Amy15A (PDE_09417, Genbank No. EPS34453.1), Amy13A (PDE_01201, Genbank No. EPS26265.1), Cel7A/CBHI (PDE_07945, Genbank No. EPS32984.1), Cel61A (PDE_05633, Genbank No. EPS30681.1), Chi18A (PDE_08122, Genbank No. EPS33160.1), Cel3A/BGLI (PDE_02736, Genbank No. EPS27792.1), Xyn10A (PDE_08094, Genbank No. EPS33132.1), Cel7B/EGI (PDE_07929, Genbank No. EPS32968.1), Cel5B/EGII (PDE_09226, Genbank No. EPS34262.1), and Cel6A/CBHII (PDE_07124, Genbank No. EPS32164.1). Their products account for 28.9, 11.0, 9.6, 5.6, 4.9, 3.4, 3.3, 2.5, 1.9, and 1.5% of the total extracellular protein of *P. oxalicum*, respectively (Liu et al., 2013b). All top 10 extracellular protein-encoding genes [Figure 4A; numbers (1) to (10) indicate the amount of secretion from highest to the lowest] were detected with significant regulation in Δ*laeA* compared with that in WT. *laeA* exhibited a strong effect on the expression at 60 h. The transcripts of *amy15A, amy13A, cel7A*/*cbh1, cel61A, chi18A, cel3A*/*bgl1, xyn10A, cel7B*/*eg1, cel5B*/*eg2*, and *cel6A*/*cbh2* detected in Δ*laeA* were only 43.2, 34.3, 13.8, 6.8, 0, 28.3, 9.5, 2.2, 32.5, and 30.5% of WT at 60 h, respectively (Figure 4B).

### LaeA not only modulates the expression of glycoside hydrolase genes but also the expression of glycoside hydrolase-related transcription factor genes

**Figure 5:**
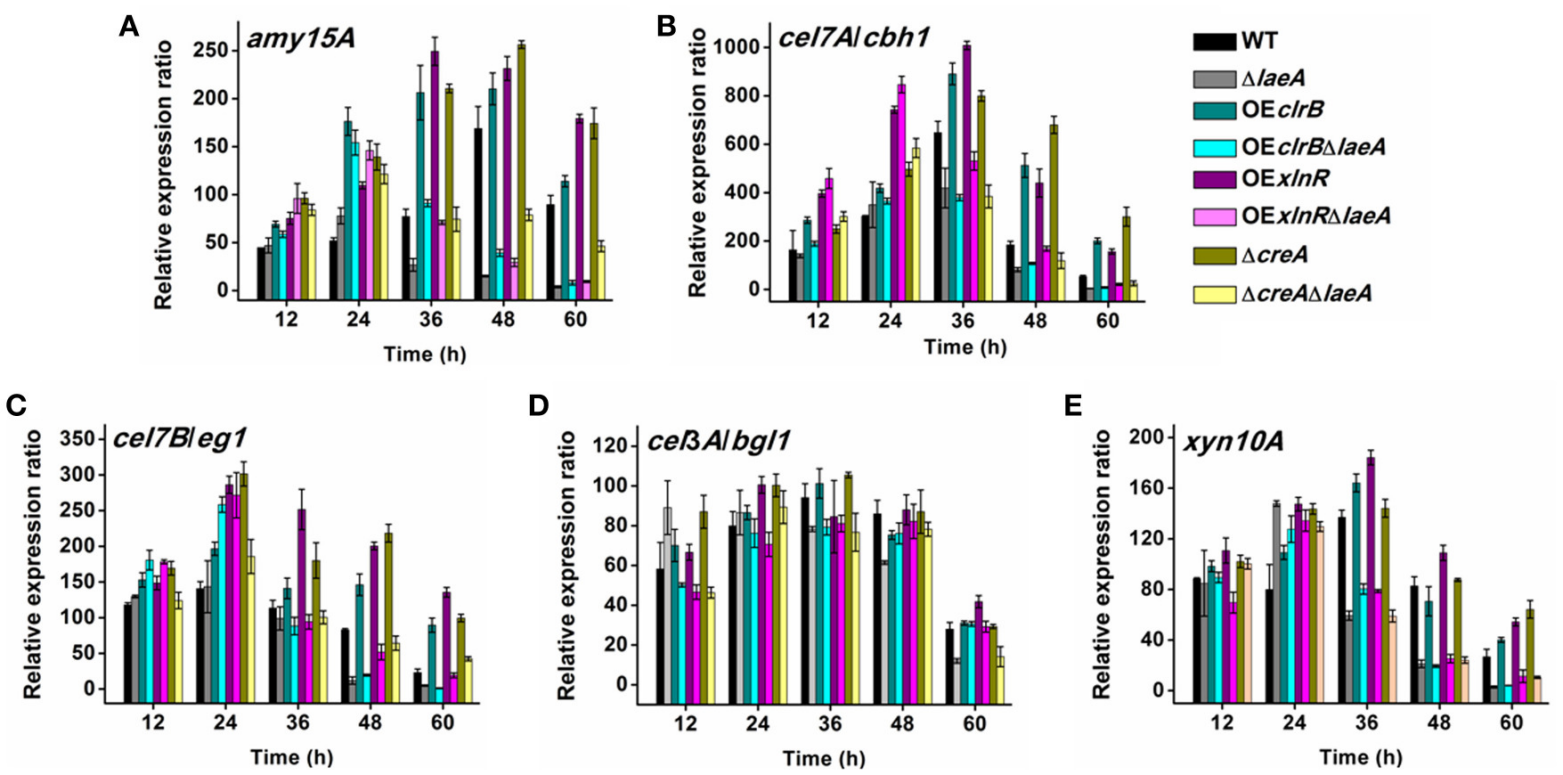
**Expression levels of prominent cellulolytic enzyme genes determined using real-time quantitative PCR (qPCR). (A)** Glucoamylase gene *amy15A*. **(B)** Cellobiohydrolase gene *cel7A/chb1*. **(C)** Endoglucanase gene *cel7B/eg1*. **(D)** β-glucosidase gene *cel3A/bgl1*. **(E)** Xylanase gene *xyn10A*.

Then, we detected the expression patterns of five out of the above 10 genes in WT and seven mutants at different culture times. These genes include the prominent glucoamylase gene *amy15A*, cellobiohydrolase gene *cel7A/chb1*, endoglucanase gene *cel7B/eg1*, β-glucosidase gene *cel3A/bgl1*, and xylanase gene *xyn10A*. The strains were pre-cultured on glucose medium for 20 h, starved for 4 h under no carbon source conditions, and transferred to cellulose and wheat bran medium for further cultivation. The transcript levels of the five genes were quantified in cells grown from 12, 24, 36, 48, and 60 h (Figure 5). The expression levels of *amy15A* (Figure 5A), *cel7A/chb1* (Figure 5B), *cel7B/eg1* (Figure 5C), and *xyn10A* (Figure 5E) in OE*clrB*, OE*xlnR*, and Δ*creA* dramatically increased from 12 to 60 h. For example, *amy15A* expression in the OE*clrB*, OE*xlnR*, and Δ*creA* increased respectively by 167, 232, and 173% at the 36 h time point compared with WT. The expression level of *cel7A/chb1* in the OE*clrB*, OE*xlnR*, and Δ*creA* increased respectively by 37, 56, and 23% compared with that of WT. However, the expression of *amy15A, cel7A/chb1, cel7B/eg1*, and *xyn10A* in Δ*laeA*, OE*clrB*Δ*laeA*, OE*xlnR*Δ*laeA*, and Δ*laeA*Δ*creA* decreased remarkably, especially in the later phases of prolonged batch cultures. At the 48 h time point, *amy15A* expression in the Δ*laeA*, OE*clrB*Δ*laeA*, OE*xlnR*Δ*laeA*, and Δ*creA*Δ*laeA* decreased to 8.93, 23.2, 17.3, and 46.4% of WT, respectively; *cel7A/chb1* expression in the Δ*laeA*, OE*clrB*Δ*laeA*, OE*xlnR*Δ*laeA*, and Δ*creA*Δ*laeA* decreased to 44.0, 58.2, 91.3, and 64.7% of WT, respectively (Figure 5).

**Figure 6:**
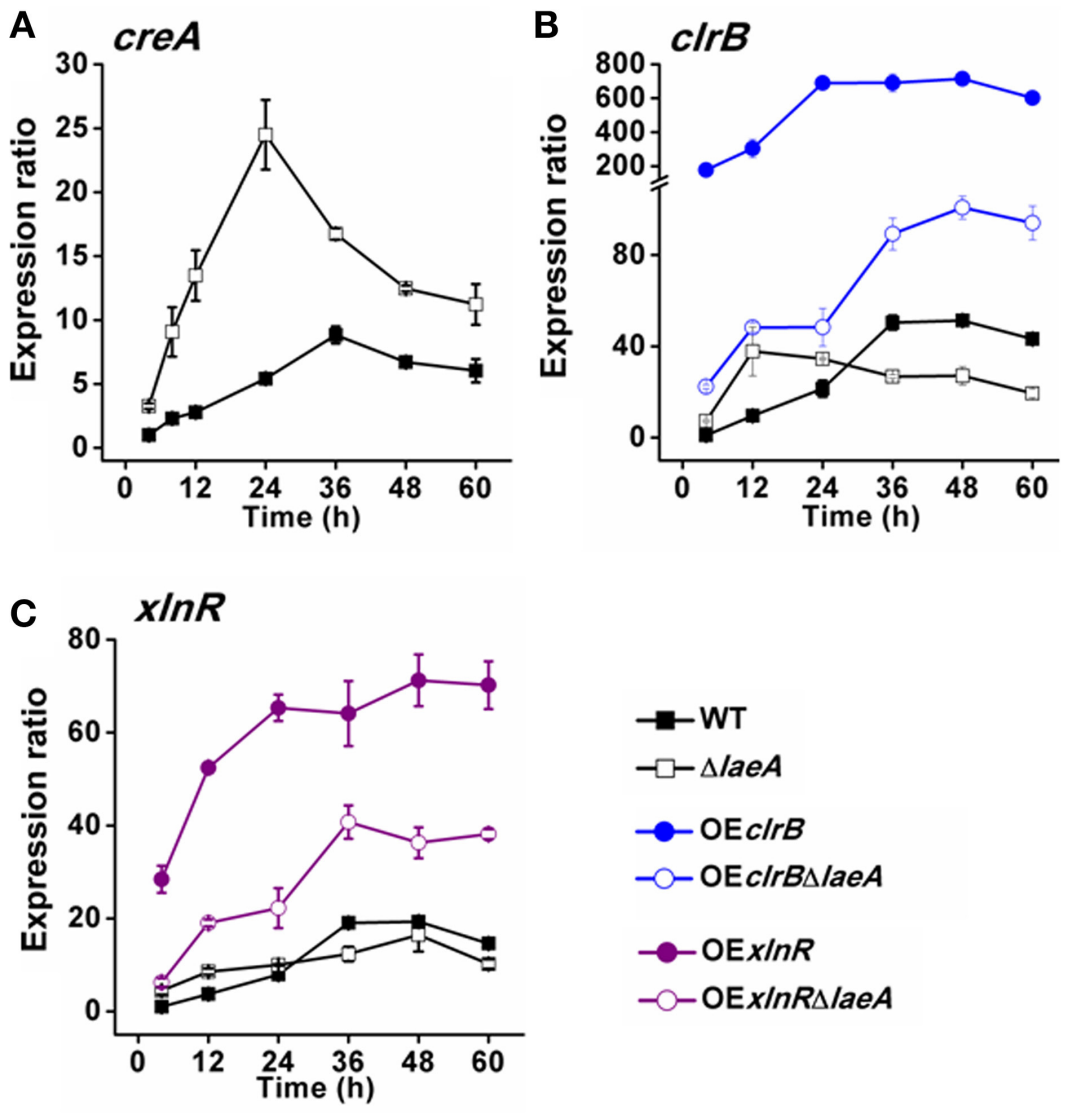
**Gene expression levels of *clrB*, *xlnR*, and *CreA* of WT and the mutants detected by qPCR. (A)** *creA*. **(B)** *clrB*. **(C)** *xlnR*.

As transcription factors *creA, clrB*, and *xlnR* have been identified to play essential roles in the expression of cellulase, and Lae1 was reported controlling transcriptional activator *xyr1* gene expression in *T. reesei* (Seiboth et al., 2012), does LaeA affect *creA, clrB*, or *xlnR* expression? Then, the transcription levels of genes *creA, clrB*, and *xlnR* were quantified in WT and the mutants grown from 12, 24, 36, 48, and 60 h (Figure 6). The expression levels of *creA* was considerably increased in Δ*laeA* compared with that in WT (Figure 6A), suggesting LaeA negative roles in regulating *creA* expression. The expression of *clrB* or *xlnR* was higher than that of in WT before 24 h-point culture time, but lower than that of in WT along with prolonged culture time (Figures 6B,C). It was expected that overexpression of *clrB* in WT (OE*clrB*) upregulate *clrB* expression remarkably; and overexpression of *xlnR* in WT (OE *xlnR*) upregulate *xlnR* expression remarkably. However, when LaeA was absent, the *clrB* expression in OE*clrB*Δ*laeA* or *xlnR* expression in OE*xlnR*Δ*laeA* decreased. The result was unexpected, because the over-expression of *clrB* or *xlnR* was driven from the *gpdA* promoter, but not the native promoters. Meanwhile, the relatively high expression of *clrB* or *xlnR* in OE*clrB*Δ*laeA* or OE*xlnR*Δ*laeA* (compared with WT) cannot rescue the impairment of cellulolytic enzyme gene expression by the deletion of *laeA* (Figure 3A), suggesting LaeA was necessary in the expression of cellulolytic enzyme gene activated by ClrB and XlnR. To investigate why *laeA* overexpression did not affect the cellulolytic enzyme production, the expression level of the key transcription factor gene (*clrB, xlnR*, and *creA*) in the OE*laeA* was compared with that of WT (Figure S5D). In contrast with that the expression of transcription repressor gene *creA* increased remarkably in Δ*laeA*, the expression levels of *creA* in OE*laeA* was identical with that of in WT. The expression of transcription activator gene *xlnR* was also identical with that of in WT. Interestingly, the expression of transcription activator gene *clrB* had a small increase (about 2-folds) compared with that of WT. Obviously, this insignificant change in *clrB* expression had no effect on cellulolytic enzyme production (Figure S5C). The results showed, normal expression of *laeA* was enough to trigger proper expression of the key transcription factor gene; excessive *laeA* expression was not needed.

### Combination of *laeA* deletion and *xlnR* overexpression activated extracellular β-xylosidase synthesis

**Figure 7:**
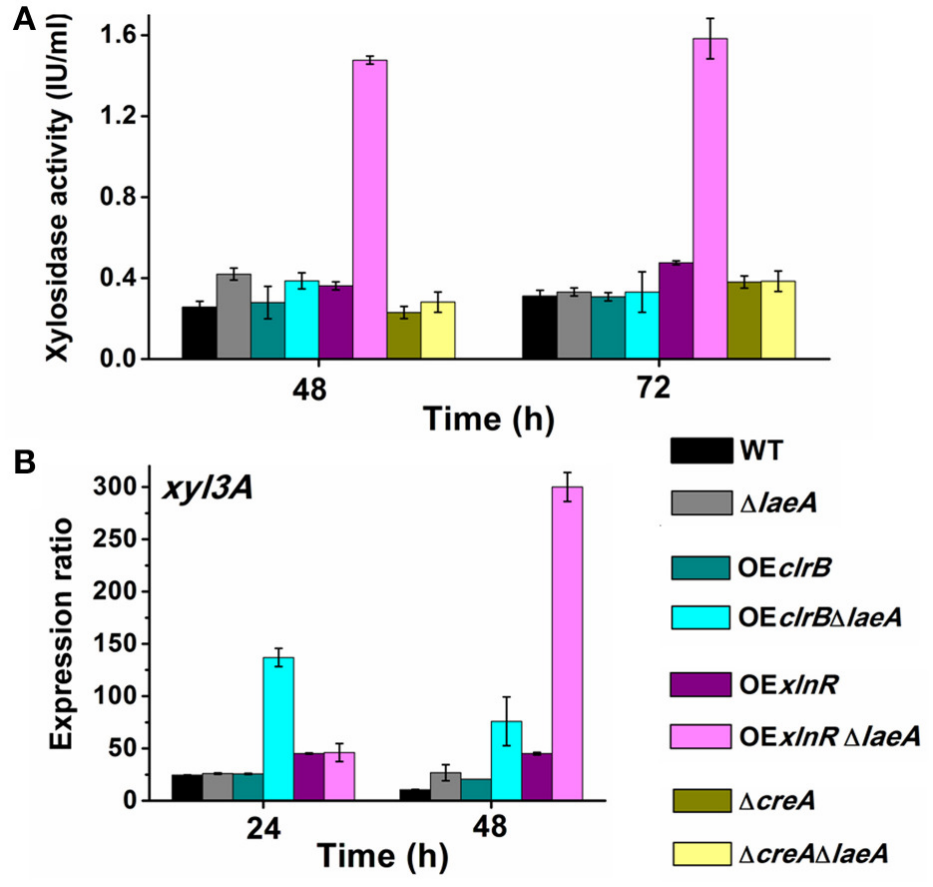
**Xylosidase activity assay and xylosidase gene expression level assay of WT and various mutants. (A)** Xylosidase activity assay. **(B)** *xyl3A* expression patterns determined by qPCR.

Of the 61 glycosidase hydrolase genes, only seven genes were upregulated at 60 h (Figure 5A, blue dots). Of the 7 genes, only PDE_00049 (a β-xylosidase-encoding gene) product was detected in *P. oxalicum* secretome (Liu et al., 2013a). Thus, the extracellular β-xylosidase activity and PDE_00049 (*xyl3A*) expression levels were assayed. Although, most cellulase and hemicellulase formation activities were reduced by the deletion of *laeA*, this deletion activated β-xylosidase synthesis, especially when *laeA* deletion and *xlnR* overexpression was combined. At 48 h, the extracellular β-xylosidase activity in OE*xlnR*Δ*laeA* was 1.5 IU/mL, which was 5.8-fold greater than that in WT (Figure 7A). The results of qPCR for *xyl3A* showed that the transcription level of OE*xlnR*Δ*laeA* was 28.5-fold greater than that of WT at 48 h. This result suggests that β-xylosidase gene expression was regulated at the transcriptional level (Figure 7B).

### No interaction was found between LaeA and transcription activators *in vitro*

Originally, we thought that transcription activators (ClrB and XlnR) might recruit LaeA playing a common regulating function, as their co-existence was required for proper cellulolytic enzyme gene expression. So, yeast two-hybrid assay was done to determine if LaeA had physical interaction with transcription factors. Yeast two-hybrid experiments did not reveal any physically interaction between LaeA and ClrB (or XlnR). On the SD/-Trp/-Leu media with X-α-gal, the strain with *laeA* and *clrB* (or *xlnR*) was not colored (Figure S7A). And They did not grown on the SD/-Trp/-Leu/-His media with 2.5 mM 3AT (Figure S7B). No interaction was observed between LaeA and CreA either.

## Discussion

LaeA is a highly conserved protein in filamentous fungi. Studies on LaeA orthologs have established significant diversity in its impact on fungal secondary metabolism and development and have been well documented in numerous fungi species (Palmer and Keller, 2010; Bayram and Braus, 2012). However, only two papers have so far reported that LaeA also participates in the expression of cellulolytic enzyme-encoding genes. LaeA is required to activate the expression of cellulase and hemicellulase gene and promote the expression of transcriptional activator Xyr1 in *T. reesei* (Seiboth et al., 2012). Moreover, the deletion of *laeA* downregulated the glucoamylase gene expression in *Aspergillus flavus* (Duran et al., 2014).

Some reports suggested asexual sporulation may be associated with glycoside hydrolase gene transcription based on the finding that the deletion of *lae1* impaired asexual sporulation and glycoside hydrolase gene transcription simultaneously; moreover, asexual sporulation triggers the expression of massive glycoside hydrolase genes (Metz et al., 2011; Seiboth et al., 2012). In the present study, we also noticed the reduced conidiation in all mutants with the deletion of *laeA*. However, sporulation and cellulase gene expression were not always coherent and sometimes occurred oppositely. Our research also indicated that the *P. oxalicum* Δ*creA* mutant with reduced conidia can form more cellulases, suggesting that the two processes are linked but not dependent on each other.

Our data showed that the deletion of *laeA* resulted in a massive reduction of glycoside hydrolase production, especially in prolonged batch cultures. The expression profiling results also showed that LaeA exhibited a stronger and wider effect on glycoside hydrolase transcriptional regulation during the stationary phase (later phases of prolonged batch cultures) than during the exponential growth phase (earlier phases of cultures). Indeed, the growth-rate dependency of LaeA function in *P*. *chrysogenum* has been reported. A significant downregulation and several morphological changes in the penicillin-biosynthesizing genes were observed in prolonged batch cultures of two different *P. chrysogenum* strains (Kosalková et al., 2009; Butchko et al., 2012). When the *laeA* deletion mutant was grown in chemostat cultures, only moderate transcriptional response and penicillin production was observed even at glucose-limited conditions (Veiga et al., 2012). Interestingly, Fekete et al. observed that the growth-rate dependence of LaeA function in *T. reesei* was in the opposite direction. The loss of function of LAE1 was more dominant at high growth rates (Fekete et al., 2014). The Velvet protein complex formed by LaeA, VeA, and VelB (Bayram et al., 2008) was assumed to play considerable roles in this process. Moreover, LAE1 was required for the expression of *vel1* (ortholog of *A. nidulans* VeA) (Karimi-Aghcheh et al., 2013). The deletion of *T. reesei lae1* or *vel1* impaired the expression of cellulases and cellulase regulator XYR1 on lactose, which suggested that cellulase expression might be regulated by the Velvet complex (Seiboth et al., 2012; Karimi-Aghcheh et al., 2014). Similarly, Hoff reported that the role of Velvet complex in *P*. *chrysogenum* was more pronounced after prolonged incubation (Hoff et al., 2010). So, as direct protein interaction has been found between *P. oxalicum* LaeA and VeA (self-communication), it was assumed the Velvet complex in *P. oxalicum* played important roles in the this process.

LaeA has been assumed to modulate transcription through histone methyltransferase functions (Reyes-Dominguez et al., 2008). However, the precise molecular function of LaeA remains unknown because its direct methyl-accepting substrate has not been identified (Patananan et al., 2013). The study on *T. reesei* showed that the LAE1-regulated expression of genes did not correspond to the changes in histone methylation; no enrichment with any of the histone marks was tested at the cellulolytic gene region or xyr1 gene locus (Seiboth et al., 2012). The precise function of LaeA toward the formation of glycoside hydrolase remained to be identified. According out research, a new found was that *creA* expression was upregulated remarkably by the deletion of *laeA*, whereas the expression *clrB* or *xlnR* was repressed by the deletion of *laeA* especially after 24 h culture time. *xyr1*/*xlnR* gene was activated with most of the cellulolytic enzyme genes in the presence of cellulose (Portnoy et al., 2011), whereas its repression was mediated by CreA (de Vries et al., 1999; Tamayo et al., 2008). The upregulation of *creA* expression by the deletion of *laeA* may have caused the repression of *xlnR* expression in the Δ*laeA* mutant. Originally, we thought the repressed expression of *clrB* and *xlnR* resulted in the downregulation of cellulolytic enzyme gene expression in Δ*laeA*, because cellulolytic enzyme gene repression also occurred in later phases of prolonged batch cultures, consistent with the tendency of *clrB* and *xlnR*. Transcription activators (ClrB and XlnR) might recruit LaeA playing a common regulating function, as their co-existence was required for proper cellulolytic enzyme gene expression. However, no physical interaction was observed between LaeA and ClrB, or XlnR, showing that LaeA and transcription activators have genetic, but physically separate functions in cellulolytic enzyme gene expression. Furthermore, the overexpression of *clrB* or *xlnR* could not rescue the impairment of cellulase gene expression caused by lack of LaeA, suggesting *clrB* or *xlnR* repression caused by *laeA* deletion was not the main reason that resulted in cellulolytic enzyme gene repression. Transcription factors ClrB, XlnR, and CreA have been identified as critical dose-dependent regulators; dose effect of transcriptional abundance is important for the high expression for cellulases (Li et al., 2015). So, we assumed that transcription repressor gene *creA* activation together with transcription activator gene *clrB* and *xlnR* repression, played synergistic and dose-controlled regulation mechanisms, which was the most important reason for cellulolytic enzyme gene repression in Δ*laeA*.

The positive effect of LaeA on the gene expression of most cellulolytic enzymes has been demonstrated clearly. However, the significant increase of xylosidase gene expression and xylosidase synthesis was unexpectedly observed in OE*xlnR*Δ*laeA*. Among the xylanolytic enzymes, endo-xylanases and β-xylosidases are important because they complete the breakdown of hemicellulose fraction (Kulkarni et al., 1999). In our study, the expression of the prominent endo-xylanase gene (*xyn10A*) and prominent β-xylosidase gene (*xyl3A*) showed distinct patterns from one another in the absence of LaeA. Although, nine xylosidase-encoding genes were predicted in the *P. oxalicum* genome (Liu et al., 2013a), PDE_00049 (*xyl3A*) product was the only extracellular β-xylosidase found in the secretome (Liu et al., 2013b), suggesting that PDE_00049 (*xyl3A*) is the most prominent and important extracellular β-xylosidase gene in *P. oxalicum*. Xyl3A belongs to glycosyl hydrolase family 3, which includes the extensively studied extracellular β-xylosidase. The deduced amino acid sequence of *P. oxalicum xyl3A* exhibited high similarities to that of *A. nidulans* XlnD (67%) (Pérez-González et al., 1998), *A. niger* XlnD (63%) (van Peij et al., 1997), and *Talaromyces emersonii* Bxl1 (68%) (Reen et al., 2003). These β-xylosidases have been verified as potentially efficient candidates to facilitate the hydrolysis of hemicellulose applications in industrial processes.

Out of the various fungi studied, Xyr1/XlnR has been extensively reported to regulate the expression of xylanolytic enzymes, such as xylanase, β-xylosidase, and D-xylose reductase (van Peij et al., 1998; Stricker et al., 2006). The constitutive expression of *xyr1*/*xlnR* can cause elevated mRNA levels of xylanase gene and β-xylosidase gene *bxl1* in *T. reesei* and *A. oryzae* (Mach-Aigner et al., 2008; Noguchi et al., 2009). Thus, the overexpression of *xlnR* was expected to upregulate the expression of *xyl3A*. However, the deletion of *laeA* unexpectedly did not downregulate the *xyl3A* expression and β-xylosidase secretion. Indeed, Duran et al. reported that the Velvet protein complex regulated various types of glycoside hydrolase expression in different patterns; the amount of glucoamylase protein was reduced, whereas the production of alpha-amylase was increased in the *veA* mutant (Duran et al., 2014), suggesting the flexibility of LaeA in regulating glycoside hydrolase gene expression. According to our previous research, ten physically linked regions of coregulated genes were found in *laeA* deletion strain, which were distributed on 5 chromosomes (Zhang et al., 2016). Among them, 8 regions were silenced and 2 regions were activated. The gene expression in these regions showed position effect. As position effect is shown to regulate expression of transgenes in *A. nidulans* (Palmer et al., 2010) due to insertion into different regions of a genome, overexpression of β-xylosidase gene by inserting it into one of the activated regions in Δ*laeA* might be an effective way to improve β-xylosidase synthesis. Furthermore, the OE*xlnR*Δ*laeA* mutant unexpectedly induced a remarkable increase in the expression of *xyl3A* and extracellular β-xylosidase synthesis. The robust induction of β-xylosidase expression and increased β-xylosidase activity in OE*xlnR*Δ*laeA* mutant was remarkably greater than that in each solo gene mutant, confirming the cumulative effects of both genes. This study confirmed that the regulation of *xyl3A* by LaeA was different from the gene regulation pathways of most cellulase or hemicellulase genes. The OE*xlnR*Δ*laeA* mutant has potential applications in the production of extracellular β-xylosidase. Indeed, the OE*xlnR*Δ*laeA* mutant has been used as the parent strain to overexpress β-xylosidase, which improved by over 20-fold compared with that of the WT (self-communication). Thus, the production of highly efficient enzymes that assist in the conversion of plant biomass is facilitated.

## Author contributions

YL, XZheng, XZhang, LB, and YZ performed the experiments. YQu, and JZ analyzed the data and revised the manuscript. YQin designed the work and drafted the manuscript.

## Funding

The National Natural Sciences Foundation of China (Grant No. 31370086); the Key research and development project of Shandong Province (2016GSF121026); the Special Project of Innovation and Achievement Transformation from Shandong Province (Grant No. 2014ZZCX09101); the China Postdoctoral Science Foundation (Grant No. 2015M582080); and the Foundation of Key Laboratory of Biofuels, Qingdao Institute of Bioenergy and Bioprocess Technology, Chinese Academy of Sciences (Grant No. CASKLB201509).

### Conflict of interest statement

The authors declare that the research was conducted in the absence of any commercial or financial relationships that could be construed as a potential conflict of interest.

## Supplementary material

Figure S1 **Strategy and results for verification of OE*laeA*, OE*clrB*Δ*laeA* and OE*xlnR*Δ*laeA*. (A)** PCR verification. Lane 1 and Lane 2, the upstream fragments amplified by ptrA-F/YlaeA-2 and downstream fragments amplified by YlaeA-1/ptrA-R to verification of OE*clrB*Δ*laeA*; lane 3 and lane 4, the upstream fragments amplified by ptrA-F/YlaeA-2 and downstream fragments amplified by YlaeA-1/ptrA-R to verification of OE*xlnR*Δ*laeA*; lane 5 and lane 6, the upstream fragments amplified by Yhyg-gpdA-F/Yhyg-gpdA-R to verification of OE*laeA*; lane 7 and lane 8, the downstream fragments amplified by YgpdA-F/YgpdA-laeA-R to verification of OE*laeA*. **(B)** Southern blot verification of OE*laeA*, OE*clrB*Δ*laeA*, and OE*xlnR*Δ*laeA*.

Figure S2 **Growth kinetics of WT and the mutants**.

Figure S3 **SDS–PAGE of extracellular protein for WT and the mutants**.

Figure S4 **Characteristics of *laeA* recomplemented strain (R*laeA*) compared with WT and Δ*laeA*. (A)** Colony morphology. **(B)** SDS–PAGE of extracellular protein. **(C)** FPA assay.

Figure S5 **Characteristics of *laeA* overexpression strain (OE*laeA*) compared with WT. (A)** Colony morphology. **(B)** SDS–PAGE of extracellular protein. **(C)** FPA assay. **(D)** Expression levels of *creA, clrB*, and *xlnR* determined using real-time quantitative PCR (qPCR).

Figure S6 **GO enrichment analysis between Δ*laeA* and WT at 24 and 60 h**. Blast2GO was used for the function enrichment analysis of gene sets with the threshold at FDR ≤ 0.05. **(A)** Functional enrichment of downregulated genes in Δ*laeA* compared with WT (4-fold or greater, FDR < 0.05). **(B)** Functional enrichment of upregulated gene in Δ*laeA* compared with WT (4-fold or greater, FDR < 0.05).

Figure S7 **Interaction analysis between LaeA and transcription factors (CreA, ClrB and XlnR) using yeast two-hybrid assay**. The open reading frames (ORFs) of the transcription factors CreA, ClrB and XlnR were amplified using cDNA from *P. oxalicum* WT as the templates and cloned into plasmid pGADT7 which containing GAL4 activation domain (AD). The open reading frames of LaeA were cloned into plasmid pGBKT7 which containing GAL4 DNA binding domain (DNA-BD). The plasmids with AD and BD (AD-*creA*, AD-*clrB*, AD-*xlnR*, and BD-*laeA*) were co-transformed into yeast AH109. **(A)** The transformants were tested on double dropout medium SD/-Trp/-Leu/ containing 10 mg/ml X-α-gal, where the positive control colony turned blue and the negative control colony keep white. The results demonstrated no direct interaction between LaeA and CreA, ClrB, or XlnR. **(B)** The transformants were tested on triple dropout medium SD/-Trp/-Leu/-His media with 2.5 mM 3AT, where the positive control and fusion proteins with interaction could growth. The results demonstrated no direct interaction between LaeA and CreA, ClrB, or XlnR. The yeast liquid was diluted multiples into gradient (10^−1^–10^−5^).

Table S1 **Primers used in construction of mutants and real-time quantitative PCR**.

Table S2 **List of downregulated genes (≥4-fold, FDR < 0.05) in Δ*laeA* when cultivated for 24 h compared with WT with significantly enriched GO terms (GO category: molecular function)**.

Table S3 **List of upregulated genes (≥4-fold, FDR < 0.05) in Δ*laeA* when cultivated for 24 h compared with WT with significantly enriched GO terms (GO category: molecular function)**.

Table S4 **List of downregulated genes (≥4-fold, FDR < 0.05) in Δ*laeA* when cultivated for 60 h compared with WT with significantly enriched GO terms (GO category: molecular function)**.
