# Supplementary figures and images for: The Different Roles of Penicillium oxalicum LaeA in the Production of Extracellular Cellulase and β-xylosidase
Source: Front Microbiol. 2016 Dec 22;7:2091. doi: 10.3389/fmicb.2016.02091 (PMC5177634; doi:10.3389/fmicb.2016.02091)

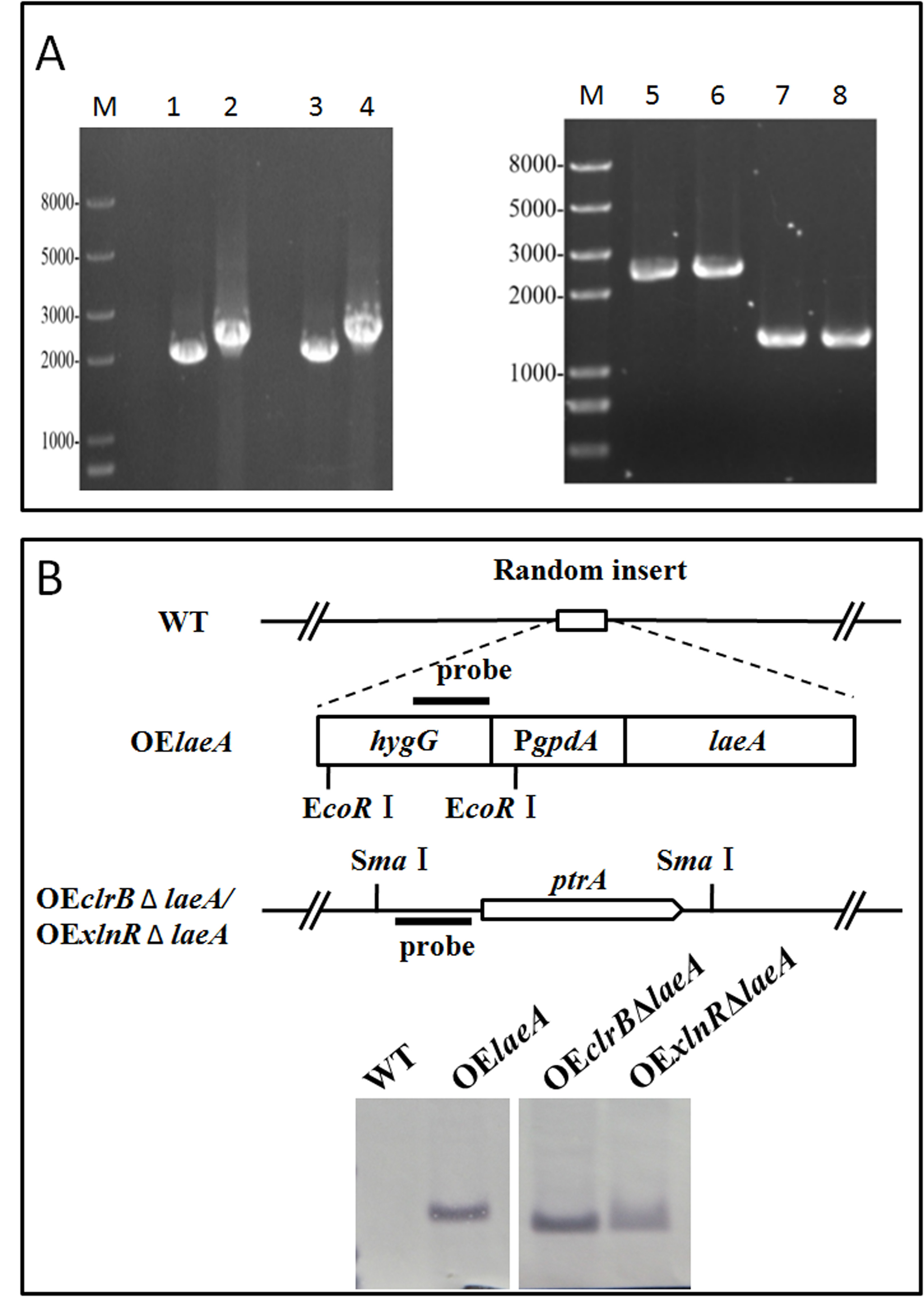

Supplement: Figure S1 — Strategy and results for verification of OElaeA, OEclrBΔlaeA and OExlnRΔlaeA. (A) PCR verification. Lane 1 and Lane 2, the upstream fragments amplified by ptrA-F/YlaeA-2 and downstream fragments amplified by YlaeA-1/ptrA-R to verification of OEclrBΔlaeA; lane 3 and lane 4, the upstream fragments amplified by ptrA-F/YlaeA-2 and downstream fragments amplified by YlaeA-1/ptrA-R to verification of OExlnRΔlaeA; lane 5 and lane 6, the upstream fragments amplified by Yhyg-gpdA-F/Yhyg-gpdA-R to verification of OElaeA; lane 7 and lane 8, the downstream fragments amplified by YgpdA-F/YgpdA-laeA-R to verification of OElaeA. (B) Southern blot verification of OElaeA, OEclrBΔlaeA, and OExlnRΔlaeA. [file Image1.JPEG]

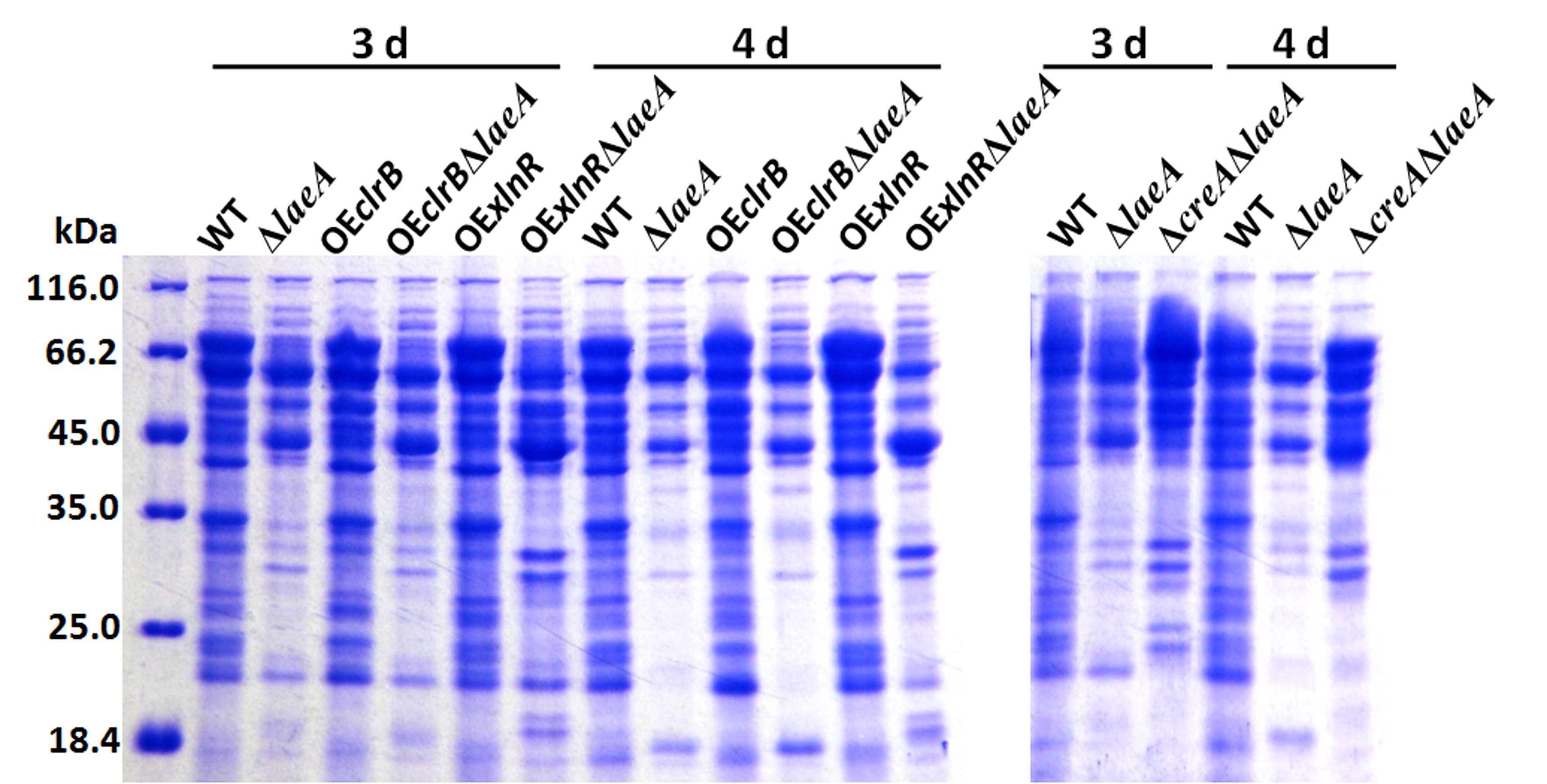

Supplement: Figure S2 — Growth kinetics of WT and the mutants. [file Image2.JPEG]

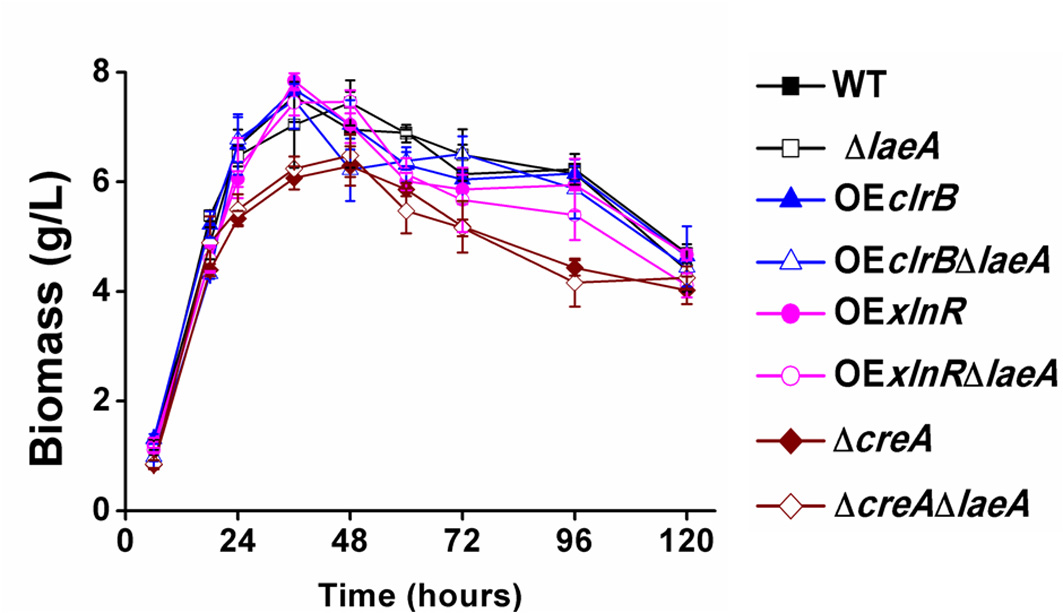

Supplement: Figure S3 — SDS–PAGE of extracellular protein for WT and the mutants. [file Image3.JPEG]

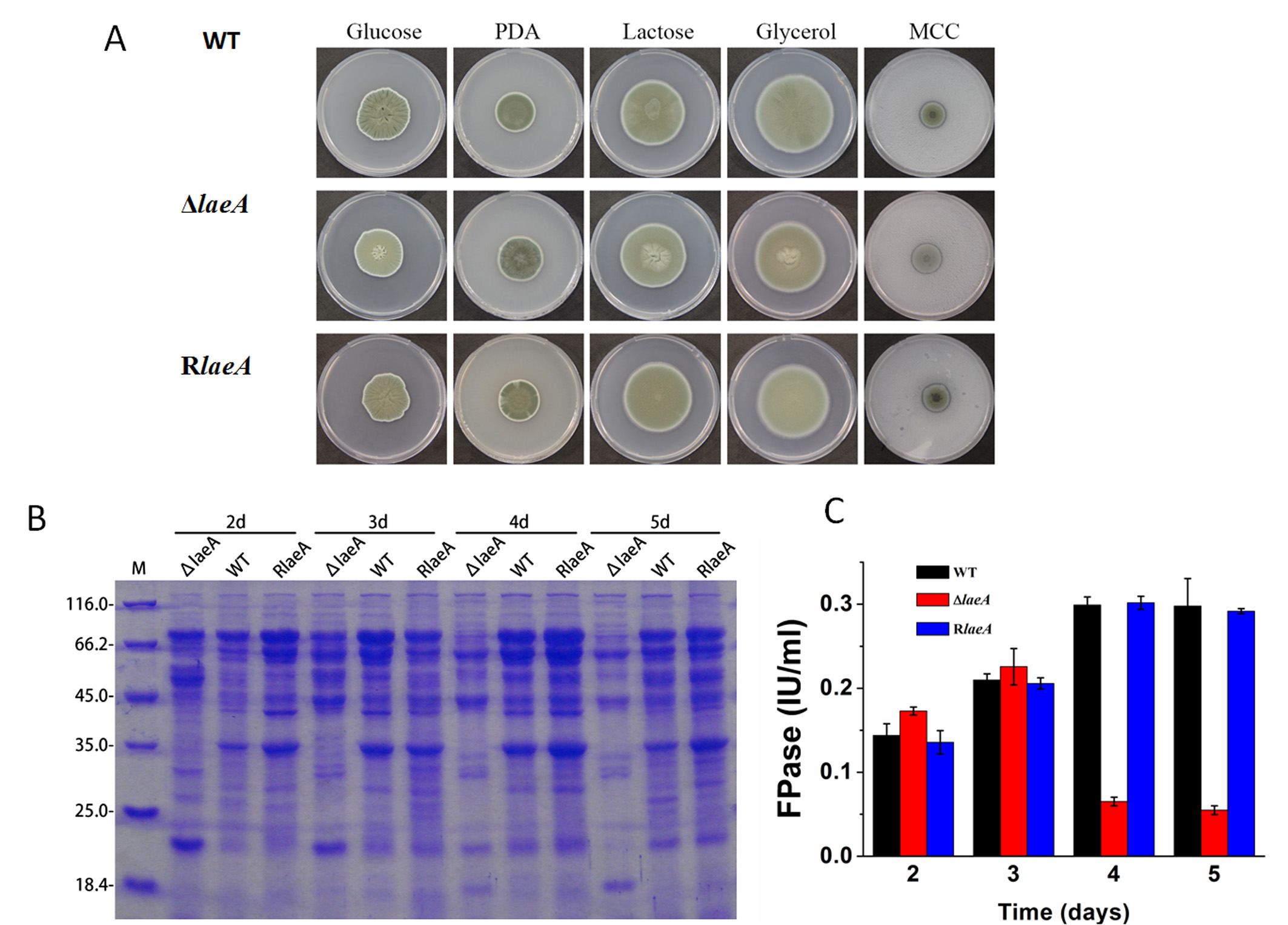

Supplement: Figure S4 — Characteristics of laeA recomplemented strain (RlaeA) compared with WT and ΔlaeA. (A) Colony morphology. (B) SDS–PAGE of extracellular protein. (C) FPA assay. [file Image4.JPEG]

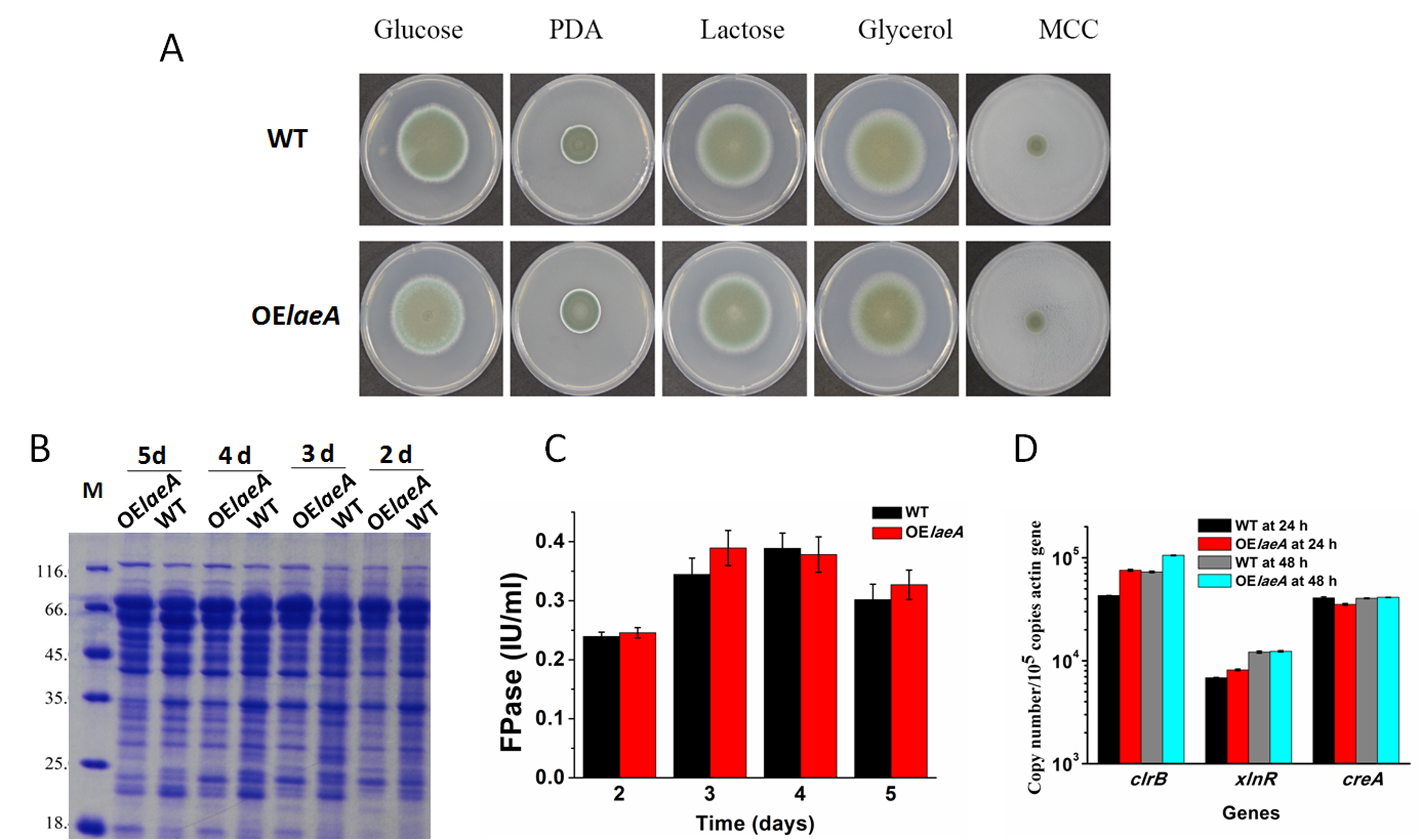

Supplement: Figure S5 — Characteristics of laeA overexpression strain (OElaeA) compared with WT. (A) Colony morphology. (B) SDS–PAGE of extracellular protein. (C) FPA assay. (D) Expression levels of creA, clrB, and xlnR determined using real-time quantitative PCR (qPCR). [file Image5.JPEG]

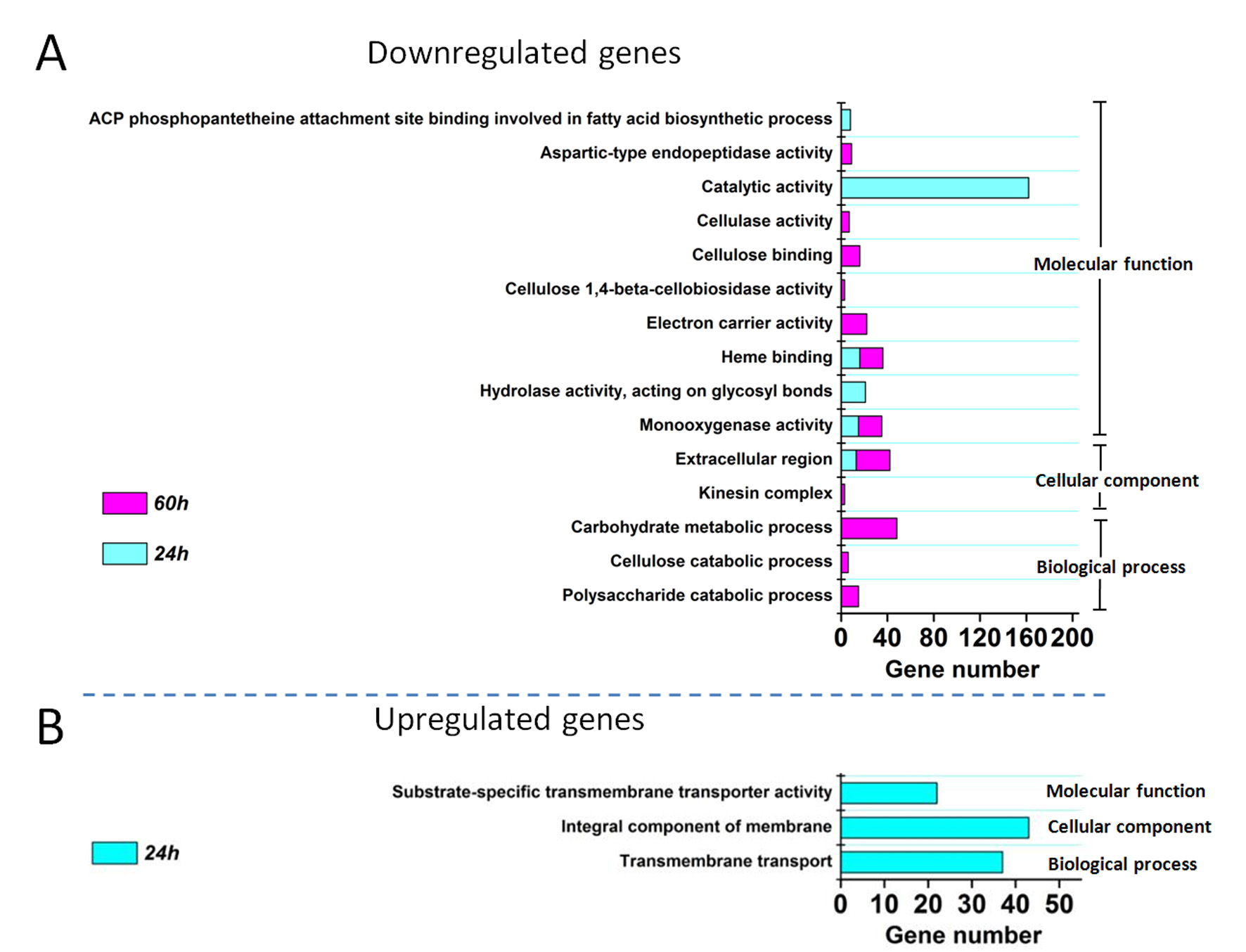

Supplement: Figure S6 — GO enrichment analysis between ΔlaeA and WT at 24 and 60 h. Blast2GO was used for the function enrichment analysis of gene sets with the threshold at FDR ≤ 0.05. (A) Functional enrichment of downregulated genes in ΔlaeA compared with WT (4-fold or greater, FDR < 0.05). (B) Functional enrichment of upregulated gene in ΔlaeA compared with WT (4-fold or greater, FDR < 0.05). [file Image6.JPEG]

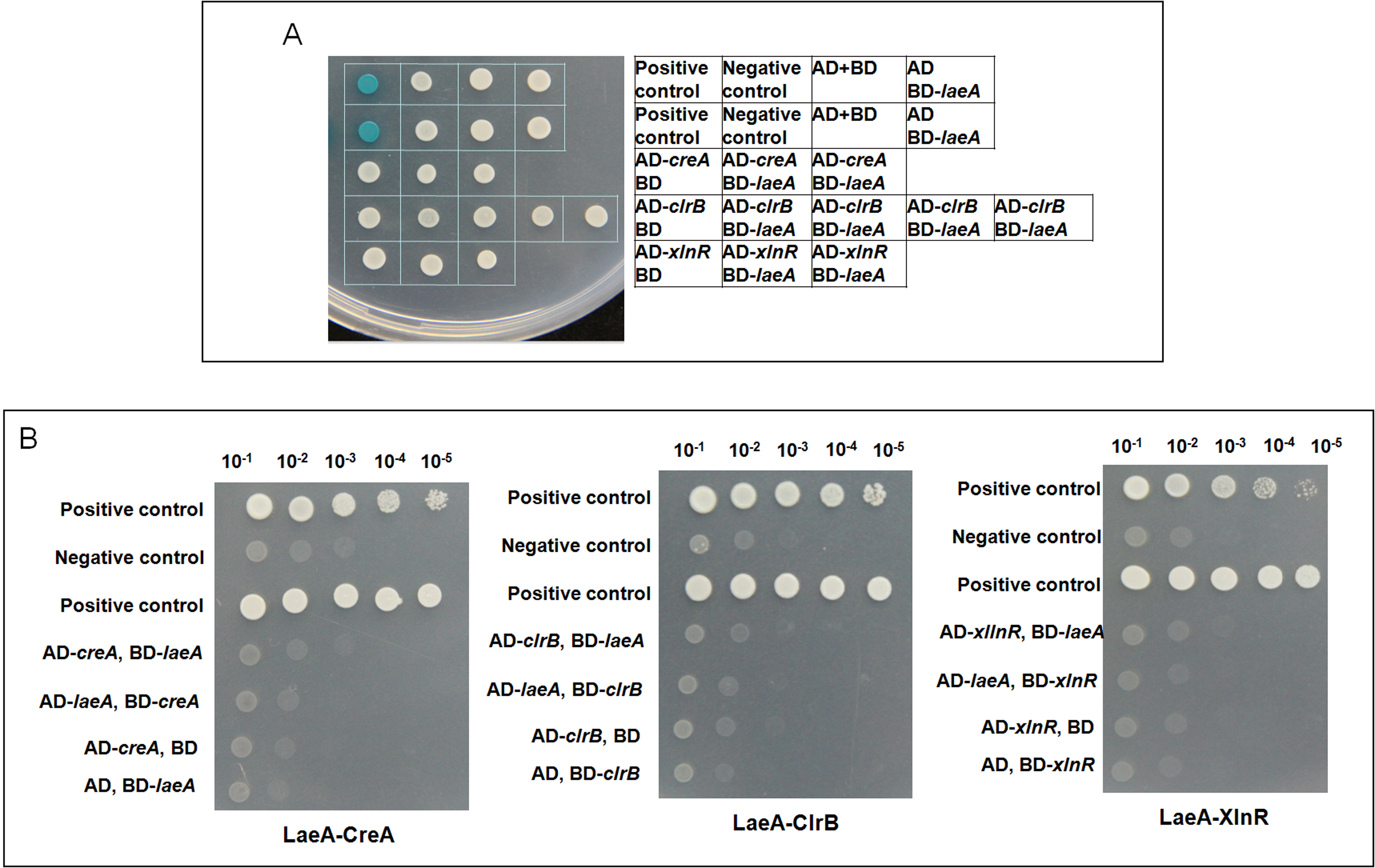

Supplement: Figure S7 — Interaction analysis between LaeA and transcription factors (CreA, ClrB and XlnR) using yeast two-hybrid assay. The open reading frames (ORFs) of the transcription factors CreA, ClrB and XlnR were amplified using cDNA from P. oxalicum WT as the templates and cloned into plasmid pGADT7 which containing GAL4 activation domain (AD). The open reading frames of LaeA were cloned into plasmid pGBKT7 which containing GAL4 DNA binding domain (DNA-BD). The plasmids with AD and BD (AD-creA, AD-clrB, AD-xlnR, and BD-laeA) were co-transformed into yeast AH109. (A) The transformants were tested on double dropout medium SD/-Trp/-Leu/ containing 10 mg/ml X-α-gal, where the positive control colony turned blue and the negative control colony keep white. The results demonstrated no direct interaction between LaeA and CreA, ClrB, or XlnR. (B) The transformants were tested on triple dropout medium SD/-Trp/-Leu/-His media with 2.5 mM 3AT, where the positive control and fusion proteins with interaction could growth. The results demonstrated no direct interaction between LaeA and CreA, ClrB, or XlnR. The yeast liquid was diluted multiples into gradient (10−1–10−5). [file Image7.JPEG]
