# Supplementary material for: The Different Roles of Penicillium oxalicum LaeA in the Production of Extracellular Cellulase and β-xylosidase
Source: Front Microbiol. 2016 Dec 22;7:2091. doi: 10.3389/fmicb.2016.02091 (PMC5177634; doi:10.3389/fmicb.2016.02091)
Supplement: Table S1 — Primers used in construction of mutants and real-time quantitative PCR. [file Table1.PDF]

Table S1 Primers used in construction of mutants and real-time quantitative PCR

Primer	Sequence (5'-3')
Primers for construction and verification of OE*laeA*, OE*clrB*Δ*laeA*, and OE*xlnR*Δ*laeA*	
hyg-F	TCTTTCCTGCGTTATCCCCTGATTC
hyg-R	CCAGGTAGGCCGAATAACTCACACAGCCAGGTAGGCCGAATAAC
gpdA-F	GTTATTCGGCCTACCTGGCTGTGTGAACTGGACGGGAAGGCACT
gpdA-LaeA-R	GATCCGTAATTGTGTAGATATCCATTTTTGCGATTGTTTGAAGTGTTCTG
laeA-F	CAGAACACTTCAAACAATCGCAAAAATGGATATCTACACAATTACGGATC
laeA-R	GGTGCCAGTGGTCATCGAACATG
UlaeA-F	CGTAAGTGGAGCCAAGGTCGATC
UlaeA-R	AATGGGATCCCGTAATCAATTGCCCGTAGCGGTCCGTAGGCGGTATTG
ptrA-F	GGGCAATTGATTACGGGATCCCATT
ptrA-R	ATGGGGTGACGATGAGCCGCTCTTG
DlaeA-F	CAAGAGCGGCTCATCGTCACCCCATCTCATGATTTATGTGTCTCAGGCTCC
DlaeA-R	GGTGCCAGTGGTCATCGAACATG
ClaeA-F	AGTACGGACTGTGTCGTTCTGCTTCA
ClaeA-R	CACCGATAAAGCATTGGATGGTTGTC
Yhyg- gpdA-F	TTGTCAAGCAAGGTAAGTGAACGAC
Yhyg-gpdA-R	GAAAGTAATAATAGTGGGTGTCGCTTGG
YgpdA-laeA-R	GATCCGTAATTGTGTAGATATCCATTTTTGCGATTGTTTGAAGTGTTCTG
YlaeA-1	CAGAAACCACGACCTTACCCTTGAG
YlaeA-2	GGGCTTGATAATCGGCGGTATGG
S-DlaeA-F	CACTTCTACACTGGCGATTCTTTCATG
S-DlaeA-R	ATAGCGGTCGTGCGGAGGGAC

S-OElaeA-F	TCTTTCCTGCGTTATCCCCTGATTC
S-OElaeA-R	CCAGGTAGGCCGAATAACTCACACAGCCAGGTAGGCCGAATAAC
Primers for real-time quantitative PCR	
act-F	CTCCATCCAGGCCGTTCTG
act-R	CATGAGGTAGTCGGTCAAGTCAC
amy15A-F	GGTCGGTTCTATTTCTCAGCTCG
amy15A-R	ACTTGGCAGGGACGGTGTAGG
clrB-F	AGCACAAGTCGAGATGGGATT
clrB-R	CGCTTGCTGGCTTCGTAAAT
cel7A-F	GTACTTGCGATCCTGATGGG
cel7A-R	CCACGGTGAAGGGAGACTTG
cel7B-F	AACCTGGAAGAACGGCACC
cel7B-R	CCTTGTCACAGTCATCGGAGC
cel3A-F	CCAACGACCCTGACTATGGC
cel3A-R	GGAACCTCGTCACCAGTAAC
creA-F	TGGGTACGAGTGAACTCCATCTT
creA-R	TGTGACCTTGACCAGGACTGTAA
laeA-F	TCTCCGCCACTGGTACACCG
laeA-R	CTCGTGTTCGTCGCTGTGC
xlnR-F	CCTGTGGATTTCCTCCGATTC
xlnR-R	CCGTCAGAAGAAGCAGGAAGC
xln10A-F	GGTCTCCAGGCTCACTTCATC
xln10A-R	GTCGAGGGCAAGTTCATACG
xyl3A-F	TACCCTGGTCAATCTGGCGG
xyl3A-R	CGCAAGTTCATATCCGTCGC
