# Supplementary material for: The Different Roles of Penicillium oxalicum LaeA in the Production of Extracellular Cellulase and β-xylosidase
Source: Front Microbiol. 2016 Dec 22;7:2091. doi: 10.3389/fmicb.2016.02091 (PMC5177634; doi:10.3389/fmicb.2016.02091)
Supplement: Table S2 — List of downregulated genes (≥4-fold, FDR < 0.05) in ΔlaeA when cultivated for 24 h compared with WT with significantly enriched GO terms (GO category: molecular function). [file Table2.PDF]

Table S2 List of downregulated genes (≥ fourfold, FDR < 0.05) in Δ*laeA* when cultivated for 24 h compared with WT with significantly enriched GO terms (GO category: molecular function)

GO-ID	Term	Gene ID (locus_tag)	Description of putative *P. oxalicum* ORF
GO:0000036	ACP phosphopantetheine attachment site binding involved in fatty acid biosynthetic process	PDE_00789	HC-toxin synthetase
		PDE_00793	HC-toxin synthetase
		PDE_00810	HC-toxin synthetase
		PDE_03926	Lovastatin nonaketide synthase
		PDE_06206	Surfactin synthase subunit 2
		PDE_07163	HC-toxin synthetase
		PDE_08155	Putative acyl-CoA synthetase yngI
		PDE_09237	Conidial yellow pigment biosynthesis polyketide synthase
GO:0003824	Catalytic activity	PDE_00013	6-hydroxy-D-nicotine oxidase
		PDE_00014	Exoglucanase 1
		PDE_00224	Pisatin demethylase
		PDE_00248	Uncharacterized protein YMR244W
		PDE_00298	Ferric/cupric reductase transmembrane component 1
		PDE_00310	Glucan 1,3-beta-glucosidase
		PDE_00317	-
		PDE_00430	UPF0586 protein C9orf41 homolog
		PDE_00584	-
		PDE_00789	HC-toxin synthetase
		PDE_00791	Uncharacterized aminotransferase C660.12c
		PDE_00793	HC-toxin synthetase
		PDE_00797	Tryptophan dimethylallyltransferase
		PDE_00799	Uncharacterized oxidoreductase ykvO
		PDE_00800	6-hydroxynicotinate 3-monooxygenase
		PDE_00802	Putative bifunctional amine oxidase DDB_G0291301
		PDE_00807	Tryptophan dimethylallyltransferase 1
		PDE_00809	6-hydroxy-D-nicotine oxidase
		PDE_00810	HC-toxin synthetase
		PDE_00840	Norsolorinic acid reductase
		PDE_00932	Trichodiene oxygenase
		PDE_01021	Probable aspartic-type endopeptidase opsB
		PDE_01023	C-5 sterol desaturase
		PDE_01080	Probable formate dehydrogenase
		PDE_01119	-
		PDE_01168	Endochitinase
		PDE_01194	Glutathione S-transferase 2
		PDE_01198	Pisatin demethylase

	PDE_01202	NADP-dependent alcohol dehydrogenase 6
	PDE_01216	Isotrichodermin C-15 hydroxylase
	PDE_01218	Multidrug resistance-associated protein 1
	PDE_01220	Fatty acid synthase subunit beta
	PDE_01315	Acid phosphatase
	PDE_01368	Sterol 24-C-methyltransferase
	PDE_01372	Phosphate-repressible acid phosphatase
	PDE_01391	Acyl-coenzyme A thioesterase 8
	PDE_01529	Pectinesterase
	PDE_01616	Probable hexaprenyl pyrophosphate synthase, mitochondrial
	PDE_01716	Acetyl-coenzyme A synthetase
	PDE_01717	Trichodiene synthase
	PDE_01718	Farnesyl pyrophosphate synthase
	PDE_01780	Putative succinate-semialdehyde dehydrogenase C1002.12c [NADP+]
	PDE_01983	Tripeptidyl-peptidase sed4
	PDE_02049	Tannase
	PDE_02061	Metacaspase-1B
	PDE_02093	Rhamnogalacturonan acetylesterase
	PDE_02114	Probable sterigmatocystin biosynthesis P450 monooxygenase stcS
	PDE_02393	L-pipecolate oxidase
	PDE_02454	Indoleamine 2,3-dioxygenase family protein
	PDE_02455	Kynureninase 2
	PDE_02514	Alpha-galactosidase 6
	PDE_02593	L-serine dehydratase
	PDE_02648	Alpha-glucosidase 2
	PDE_02682	Endo-1,4-beta-xylanase A
	PDE_02759	Epoxide hydrolase 3
	PDE_03003	Probable DNA primase large subunit
	PDE_03134	Amino-acid acetyltransferase, mitochondrial
	PDE_03296	N,O-diacetylmuramidase
	PDE_03322	FAD-containing monooxygenase EthA
	PDE_03399	Acid phosphatase
	PDE_03634	Uncharacterized protein ydhS
	PDE_03926	Lovastatin nonaketide synthase
	PDE_03993	Probable mitochondrial chaperone BCS1-B
	PDE_04039	Ornithine decarboxylase
	PDE_04162	Endopolygalacturonase AN8327
	PDE_04182	Acetylxylan esterase 2
	PDE_04378	Phosphoglycolate phosphatase
	PDE_04393	Hippurate hydrolase
	PDE_04566	Epoxide hydrolase 1

	PDE_04604	Uncharacterized oxidoreductase yxbG
	PDE_04605	Salicylate hydroxylase
	PDE_04681	Phospholipase C 3
	PDE_05002	Acetamidase
	PDE_05138	Probable phosphoketolase
	PDE_05156	NADP-dependent alcohol dehydrogenase 6
	PDE_05223	Glucan endo-1,3-alpha-glucosidase agn1
	PDE_05238	tRNA 2'-phosphotransferase 1
	PDE_05305	Serine-type carboxypeptidase F
	PDE_05369	Tyrosine-protein phosphatase pmp1
	PDE_05422	Uncharacterized protein yxjG
	PDE_05506	Probable diacylglycerol pyrophosphate phosphatase 1
	PDE_05592	Uncharacterized helicase C694.02
	PDE_05737	-
	PDE_05885	Indoleamine 2,3-dioxygenase 2
	PDE_05886	Cytochrome P450 1A1
	PDE_05889	-
	PDE_05931	Rhamnogalacturonase A
	PDE_05951	Arylamine N-acetyltransferase
	PDE_06013	Probable squalene synthase
	PDE_06059	4-coumarate--CoA ligase-like 5
	PDE_06069	6-hydroxynicotinate 3-monooxygenase
	PDE_06154	-
	PDE_06206	Surfactin synthase subunit 2
	PDE_06220	DNA polymerase epsilon subunit B
	PDE_06246	Kinesin light chain
	PDE_06327	Isotrichodermin C-15 hydroxylase
	PDE_06340	Non-hemolytic phospholipase C
	PDE_06352	-
	PDE_06389	Oxalate decarboxylase oxdD
	PDE_06451	-
	PDE_06558	-
	PDE_06566	Killer toxin subunits alpha/beta
	PDE_06631	Probable endo-1,3(4)-beta-glucanase AO090023000083
	PDE_06814	Probable acid phosphatase
	PDE_06887	Tetracycline resistance protein from transposon Tn4351/Tn4400
	PDE_06951	1,3-beta-glucanosyltransferase gel4
	PDE_06972	Ent-kaurene oxidase
	PDE_07085	Protein GCY
	PDE_07163	HC-toxin synthetase
	PDE_07165	Metal resistance protein YCF1

	PDE_07204	Tyrosine-protein phosphatase
	PDE_07205	Geranylgeranyl pyrophosphate synthase
	PDE_07206	Isotrichodermin C-15 hydroxylase
	PDE_07208	Short-chain dehydrogenase/reductase family 16C member 6
	PDE_07226	4-hydroxyacetophenone monooxygenase
	PDE_07344	Aspergillopepsin-F
	PDE_07585	Arabinan endo-1,5-alpha-L-arabinosidase
	PDE_07724	Putative aspergillopepsin A-like aspartic endopeptidase AFUA_2G15950
	PDE_07886	Probable proline dehydrogenase, mitochondrial
	PDE_07895	Uncharacterized methyltransferase C1B3.06c
	PDE_07930	Probable aspartic-type endopeptidase opsB
	PDE_07938	Polygalacturonase
	PDE_08103	Tryptophan synthase
	PDE_08122	Endochitinase 1
	PDE_08127	Ubiquitin carboxyl-terminal hydrolase isozyme L3
	PDE_08143	UPF0012 hydrolase C26A3.11
	PDE_08155	Putative acyl-CoA synthetase yngI
	PDE_08156	1-aminocyclopropane-1-carboxylate oxidase
	PDE_08196	Mitogen-activated protein kinase
	PDE_08298	Aldehyde dehydrogenase family 3 member H1
	PDE_08415	Putative diaminopropionate ammonia-lyase
	PDE_08598	Sphingomyelin phosphodiesterase B
	PDE_08660	Isotrichodermin C-15 hydroxylase
	PDE_08698	Sporulation protein kinase pit1
	PDE_08742	Interferon-induced GTP-binding protein Mx1
	PDE_08743	-
	PDE_08747	-
	PDE_08804	Probable beta-mannosidase A
	PDE_08919	Cell division control protein 2
	PDE_09019	Probable beta-glucosidase F
	PDE_09229	3-hydroxybenzoate 6-hydroxylase 1
	PDE_09230	Probable cytochrome P450 6a13
	PDE_09231	Uncharacterized oxidoreductase C663.08c
	PDE_09234	Putative sterigmatocystin biosynthesis protein stcQ
	PDE_09236	Scytalone dehydratase
	PDE_09237	Conidial yellow pigment biosynthesis polyketide synthase
	PDE_09238	Beta-lactamase-like protein 2
	PDE_09239	Versicolorin reductase

		PDE_09242	Probable S-adenosylmethionine-dependent methyltransferase CRG1
		PDE_09250	-
		PDE_09353	BAHD acyltransferase DCR
		PDE_09380	Uncharacterized oxidoreductase yusZ
		PDE_09467	Signal peptidase complex subunit SPC3
		PDE_09532	Endochitinase 2
		PDE_09616	Alcohol dehydrogenase
		PDE_09758	Phenol 2-monooxygenase
		PDE_09759	Aromatic amino acid aminotransferase C56E4.03
		PDE_09761	Probable 4-hydroxyphenylpyruvate dioxygenase 2
		PDE_09762	Cytochrome b2, mitochondrial
		PDE_09810	Uncharacterized protein C106.17c
		PDE_09910	Methylisocitrate lyase
		PDE_10011	Glucan endo-1,3-alpha-glucosidase agn1
GO:0020037	Heme binding	PDE_00224	Pisatin demethylase
		PDE_00808	Cytochrome P450 83B1
		PDE_00811	Ent-kaurene oxidase
		PDE_00932	Trichodiene oxygenase
		PDE_01198	Pisatin demethylase
		PDE_01216	Isotrichodermin C-15 hydroxylase
		PDE_02114	Probable sterigmatocystin biosynthesis P450 monooxygenase stcS
		PDE_02454	Indoleamine 2,3-dioxygenase family protein
		PDE_05885	Indoleamine 2,3-dioxygenase 2
		PDE_05886	Cytochrome P450 1A1
		PDE_06327	Isotrichodermin C-15 hydroxylase
		PDE_06972	Ent-kaurene oxidase
		PDE_07206	Isotrichodermin C-15 hydroxylase
		PDE_08660	Isotrichodermin C-15 hydroxylase
		PDE_09230	Probable cytochrome P450 6a13
		PDE_09762	Cytochrome b2, mitochondrial
GO:0016798	Hydrolase activity, acting on glycosyl bonds	PDE_00014	Exoglucanase 1
		PDE_00248	Uncharacterized protein YMR244W
		PDE_00310	Glucan 1,3-beta-glucosidase
		PDE_01168	Endochitinase
		PDE_02514	Alpha-galactosidase 6
		PDE_02648	Alpha-glucosidase 2
		PDE_02682	Endo-1,4-beta-xylanase A
		PDE_03296	N,O-diacetylmuramidase
		PDE_04162	Endopolygalacturonase AN8327
		PDE_04182	Acetylxylan esterase 2

		PDE_04393	Hippurate hydrolase
		PDE_05223	Glucan endo-1,3-alpha-glucosidase agn1
		PDE_05931	Rhamnogalacturonase A
		PDE_06451	-
		PDE_06566	Killer toxin subunits alpha/beta
		PDE_06631	Probable endo-1,3(4)-beta-glucanase AO090023000083
		PDE_07938	Polygalacturonase
		PDE_08122	Endochitinase 1
		PDE_08804	Probable beta-mannosidase A
		PDE_09019	Probable beta-glucosidase F
		PDE_09532	Endochitinase 2
GO:0004497	Monooxygenase activity	PDE_00224	Pisatin demethylase
		PDE_00932	Trichodiene oxygenase
		PDE_01198	Pisatin demethylase
		PDE_01216	Isotrichodermin C-15 hydroxylase
		PDE_02114	Probable sterigmatocystin biosynthesis P450 monooxygenase stcS
		PDE_03322	FAD-containing monooxygenase EthA
		PDE_04605	Salicylate hydroxylase
		PDE_05886	Cytochrome P450 1A1
		PDE_06069	6-hydroxynicotinate 3-monooxygenase
		PDE_06887	Tetracycline resistance protein from transposon Tn4351/Tn4400
		PDE_06972	Ent-kaurene oxidase
		PDE_07206	Isotrichodermin C-15 hydroxylase
		PDE_07226	4-hydroxyacetophenone monooxygenase
		PDE_09229	3-hydroxybenzoate 6-hydroxylase 1
		PDE_09230	Probable cytochrome P450 6a13
