# Supplementary material for: The Different Roles of Penicillium oxalicum LaeA in the Production of Extracellular Cellulase and β-xylosidase
Source: Front Microbiol. 2016 Dec 22;7:2091. doi: 10.3389/fmicb.2016.02091 (PMC5177634; doi:10.3389/fmicb.2016.02091)
Supplement: Table S3 — List of upregulated genes (≥4-fold, FDR < 0.05) in ΔlaeA when cultivated for 24 h compared with WT with significantly enriched GO terms (GO category: molecular function). [file Table3.PDF]

Table S3 List of upregulated genes (≥ fourfold, FDR < 0.05) in Δ*laeA* when cultivated for 24 h compared with WT with significantly enriched GO terms (GO category: molecular function)

**GO-ID**	**Term**	**Gene ID (locus_tag)**	**Description of putative *P. oxalicum* ORF**
GO:0022891	Substrate-specific transmembrane transporter activity	PDE_00156	Uncharacterized permease C29B12.14c
		PDE_00197	High-affinity fructose transporter ght6
		PDE_00611	Quinate permease
		PDE_00845	General amino-acid permease GAP1
		PDE_01144	ATP synthase subunit alpha, mitochondrial
		PDE_01145	V-type proton ATPase catalytic subunit A
		PDE_01211	General amino acid permease AGP2
		PDE_01388	Probable glucose transporter rco-3
		PDE_02170	Putative HC-toxin efflux carrier TOXA
		PDE_02574	Quinate permease
		PDE_02875	Vacuolar calcium ion transporter
		PDE_03438	-
		PDE_03475	Probable glucose transporter rco-3
		PDE_04131	High-affinity glucose transporter
		PDE_04634	Calcium-transporting ATPase 3
		PDE_05051	Putative copper-transporting ATPase 3
		PDE_05150	Calcium-transporting ATPase 2
		PDE_08756	Copper transport protein ctr4
		PDE_09323	GABA-specific permease
		PDE_09395	Lactose permease
		PDE_09534	Lactose permease
		PDE_09628	Sugar transporter STL1
