# Supplementary material for: The Different Roles of Penicillium oxalicum LaeA in the Production of Extracellular Cellulase and β-xylosidase
Source: Front Microbiol. 2016 Dec 22;7:2091. doi: 10.3389/fmicb.2016.02091 (PMC5177634; doi:10.3389/fmicb.2016.02091)
Supplement: Table S4 — List of downregulated genes (≥4-fold, FDR < 0.05) in ΔlaeA when cultivated for 60 h compared with WT with significantly enriched GO terms (GO category: molecular function). [file Table4.PDF]

Table S4 List of downregulated genes (≥ fourfold, FDR < 0.05) in Δ*laeA* when cultivated for 60 h compared with WT with significantly enriched GO terms (GO category: molecular function)

**GO-ID**	**Term**	**Gene ID (locus_tag)**	**Description of putative *P. oxalicum* ORF**
GO:0004190	Aspartic-type endopeptidase activity	PDE_01217	Fatty acid synthase subunit alpha
		PDE_01852	Probable aspartic-type endopeptidase OPSB
		PDE_06014	Aspergillopepsin-2
		PDE_07110	Aspergillopepsin
		PDE_07344	Aspergillopepsin-F
		PDE_07927	Penicillopepsin
		PDE_07933	Aspergillopepsin-2
		PDE_08305	Putative aspergillopepsin A-like aspartic endopeptidase AFUA_2G15950
GO:0008810	Cellulase activity	PDE_00507	Endoglucanase EG-II
		PDE_00698	Endoglucanase-4
		PDE_01261	Endoglucanase-4
		PDE_05633	Endoglucanase-4
		PDE_07124	Probable 1,4-beta-D-glucan cellobiohydrolase C
		PDE_07928	Endoglucanase-5
		PDE_07929	Endoglucanase EG-1
GO:0016162	Cellulose 1,4-beta-cellobiosidase activity	PDE_05445	Probable 1,4-beta-D-glucan cellobiohydrolase A
		PDE_07124	Probable 1,4-beta-D-glucan cellobiohydrolase C
		PDE_07945	Exoglucanase 1
GO:0030248	Cellulose binding	PDE_00015	Putative beta-xylosidase
		PDE_00016	Alpha-L-arabinofuranosidase axhA-2
		PDE_00507	Endoglucanase EG-II
		PDE_01261	Endoglucanase-4
		PDE_02101	Probable endo-1,4-beta-xylanase B
		PDE_02102	Expansin-B1
		PDE_02514	Alpha-galactosidase 6
		PDE_02682	Endo-1,4-beta-xylanase A
		PDE_04182	Acetylxylan esterase 2
		PDE_05445	Probable 1,4-beta-D-glucan cellobiohydrolase A
		PDE_06023	Probable mannan endo-1,4-beta-mannosidase F
		PDE_06649	Probable feruloyl esterase C
		PDE_07124	Probable 1,4-beta-D-glucan cellobiohydrolase C
		PDE_07928	Endoglucanase-5
		PDE_07929	Endoglucanase EG-1
		PDE_07945	Exoglucanase 1
GO:0009055	Electron carrier activity	PDE_00224	Pisatin demethylase
		PDE_00808	Cytochrome P450 83B1
		PDE_00811	Ent-kaurene oxidase
		PDE_00932	Trichodiene oxygenase

		PDE_01198	Pisatin demethylase
		PDE_01216	Isotrichodermin C-15 hydroxylase
		PDE_02121	Ent-kaurene oxidase
		PDE_02656	Cytochrome P450 52A13
		PDE_03360	Ferric/cupric reductase transmembrane component 7
		PDE_05886	Cytochrome P450 1A1
		PDE_06327	Isotrichodermin C-15 hydroxylase
		PDE_06706	Psi-producing oxygenase C
		PDE_06793	Monothiol glutaredoxin-7
		PDE_06972	Ent-kaurene oxidase
		PDE_07206	Isotrichodermin C-15 hydroxylase
		PDE_07564	Thioredoxin
		PDE_08125	Cytochrome b-245 heavy chain
		PDE_08660	Isotrichodermin C-15 hydroxylase
		PDE_08821	Cytochrome P450 97B3, chloroplastic
		PDE_09230	Probable cytochrome P450 6a13
		PDE_09748	Trichodiene oxygenase
		PDE_10010	Averantin oxidoreductase
GO:0020037	Heme binding	PDE_00224	Pisatin demethylase
		PDE_00808	Cytochrome P450 83B1
		PDE_00811	Ent-kaurene oxidase
		PDE_00932	Trichodiene oxygenase
		PDE_01198	Pisatin demethylase
		PDE_01216	Isotrichodermin C-15 hydroxylase
		PDE_02121	Ent-kaurene oxidase
		PDE_02406	Cytochrome b2, mitochondrial
		PDE_02656	Cytochrome P450 52A13
		PDE_05886	Cytochrome P450 1A1
		PDE_06327	Isotrichodermin C-15 hydroxylase
		PDE_06706	Psi-producing oxygenase C
		PDE_06972	Ent-kaurene oxidase
		PDE_07206	Isotrichodermin C-15 hydroxylase
		PDE_08660	Isotrichodermin C-15 hydroxylase
		PDE_08821	Cytochrome P450 97B3, chloroplastic
		PDE_09230	Probable cytochrome P450 6a13
		PDE_09748	Trichodiene oxygenase
		PDE_09762	Cytochrome b2, mitochondrial
		PDE_10010	Averantin oxidoreductase
GO:0004497	Monooxygenase activity	PDE_00224	Pisatin demethylase
		PDE_00932	Trichodiene oxygenase
		PDE_01198	Pisatin demethylase
		PDE_01216	Isotrichodermin C-15 hydroxylase
		PDE_02118	3-hydroxybenzoate 6-hydroxylase 1

	PDE_02121	Ent-kaurene oxidase
	PDE_02656	Cytochrome P450 52A13
	PDE_03468	Putative sterigmatocystin biosynthesis monooxygenase stcW
	PDE_04264	-
	PDE_04605	Salicylate hydroxylase
	PDE_05886	Cytochrome P450 1A1
	PDE_05952	Salicylate hydroxylase
	PDE_06706	Psi-producing oxygenase C
	PDE_06972	Ent-kaurene oxidase
	PDE_07206	Isotrichodermin C-15 hydroxylase
	PDE_08821	Cytochrome P450 97B3, chloroplastic
	PDE_09229	3-hydroxybenzoate 6-hydroxylase 1
	PDE_09230	Probable cytochrome P450 6a13
	PDE_09748	Trichodiene oxygenase
	PDE_09911	Salicylate hydroxylase
